# Supplementary material for: Opt-out rates and reasons for non-participation in a single-arm feasibility trial (ENGAGE) of a guided internet-administered CBT-based intervention for parents of children treated for cancer: a nested cross-sectional survey
Source: BMJ Open. 2022 Apr 1;12(4):e056758. doi: 10.1136/bmjopen-2021-056758 (PMC8977820; doi:10.1136/bmjopen-2021-056758)
Supplement: Author's manuscript [file bmjopen-2021-056758.draft_revisions.pdf]

BMJ Open

BMJ Open is committed to open peer review. As part of this commitment we make the peer review history of every article we publish publicly available.

When an article is published we post the peer reviewers' comments and the authors' responses online. We also post the versions of the paper that were used during peer review. These are the versions that the peer review comments apply to.

The versions of the paper that follow are the versions that were submitted during the peer review process. They are not the versions of record or the final published versions. They should not be cited or distributed as the published version of this manuscript.

BMJ Open is an open access journal and the full, final, typeset and author-corrected version of record of the manuscript is available on our site with no access controls, subscription charges or pay-per-view fees (http://bmjopen.bmj.com).

If you have any questions on BMJ Open's open peer review process please email info.bmjopen@bmj.com

BMJ Open

Opt-out rates and reasons for non-participation in a single-arm feasibility study of a guided internet-administered CBT-based intervention for parents of children treated for cancer: a nested cross-sectional survey

Journal:	*BMJ Open*
Manuscript ID	bmjopen-2021-056758
Article Type:	Original research
Date Submitted by the Author:	24-Aug-2021

Complete List of Authors:	Hagström, Josefin; Uppsala University, Healthcare Sciences and e-Health, Department of Women's and Children's Health<br>Woodford, Joanne; Uppsala University, Healthcare Sciences and e-Health, Department of Women´s and Children´s Health<br>von Essen, Agnes; Uppsala University, Healthcare Sciences and e-Health, Department of Women's and Children's Health<br>Lähteenmäki, Paivi ; TYKS Turku University Hospital Department of Paediatrics and Adolescent Medicine; Karolinska Institute, Pediatric Oncology and Pediatric Surgery, Department of Women's and Children's Health<br>von Essen, Louise; Uppsala University, Healthcare Sciences and e-Health, Department of Women's and Children's Health

Keywords:	Depression & mood disorders < PSYCHIATRY, STATISTICS & RESEARCH METHODS, QUALITATIVE RESEARCH, Paediatric oncology < ONCOLOGY

SCHOLARONE™
Manuscripts

I, the Submitting Author has the right to grant and does grant on behalf of all authors of the Work (as defined in the below author licence), an exclusive licence and/or a non-exclusive licence for contributions from authors who are: i) UK Crown employees; ii) where BMJ has agreed a CC-BY licence shall apply, and/or iii) in accordance with the terms applicable for US Federal Government officers or employees acting as part of their official duties; on a worldwide, perpetual, irrevocable, royalty-free basis to BMJ Publishing Group Ltd ("BMJ") its licensees and where the relevant Journal is co-owned by BMJ to the co-owners of the Journal, to publish the Work in this journal and any other BMJ products and to exploit all rights, as set out in our licence.

The Submitting Author accepts and understands that any supply made under these terms is made by BMJ to the Submitting Author unless you are acting as an employee on behalf of your employer or a postgraduate student of an affiliated institution which is paying any applicable article publishing charge ("APC") for Open Access articles. Where the Submitting Author wishes to make the Work available on an Open Access basis (and intends to pay the relevant APC), the terms of reuse of such Open Access shall be governed by a Creative Commons licence – details of these licences and which Creative Commons licence will apply to this Work are set out in our licence referred to above.

Other than as permitted in any relevant BMJ Author's Self Archiving Policies, I confirm this Work has not been accepted for publication elsewhere, is not being considered for publication elsewhere and does not duplicate material already published. I confirm all authors consent to publication of this Work and authorise the granting of this licence.

For peer review only

Opt-out rates and reasons for non-participation in a single-arm feasibility study of a guided internet-administered CBT-based intervention for parents of children treated for cancer: a nested cross-sectional survey

Josefin HAGSTRÖM[1#] (josefin.hagstrom@kbh.uu.se); Joanne WOODFORD[1#] (joanne.woodford@kbh.uu.se); Agnes von ESSEN[1] (agnes-von.essen@kbh.uu.se); Päivi LÄHTEENMÄKI[2,3] (paivi.lahteenmaki@ki.se); Louise von ESSEN[1*] (louise-von.essen@kbh.uu.se).

[#]Joint authorship

Corresponding author

*Professor Louise von Essen, Healthcare Sciences and e-Health, Department of Women's and Children's Health, Uppsala University. Dag Hammarskjölds väg 14B, 751 05 Uppsala, Sweden. e-mail: louise.von.essen@kbh.uu.se Telephone: +46 070-425 07 14

Affiliation

[1]Healthcare Sciences and e-Health, Department of Women´s and Children´s Health, Uppsala University, Uppsala, Sweden

[2]Department of Pediatric and Adolescent Medicine, Turku University Hospital and Turku University, Turku, Finland

[3]Pediatric Oncology and Pediatric Surgery, Department of Women's and Children's Health, Karolinska Institutet, Stockholm, Sweden

Word Count: 5049

For peer review only

ABSTRACT

Objectives: Primary objectives examined opt-out and consent rates, mode and time point of opt-out, and sociodemographic characteristics of those who opted out versus chose to participate in a single-arm feasibility study (ENGAGE) of a guided, internet-administered, CBT-based intervention for parents of children treated for cancer. Secondary objectives examined reasons for non-participation.

Design: A cross-sectional survey nested within the ENGAGE study.

Setting: The intervention was delivered from Uppsala University, with participants located throughout Sweden.

Participants: Potential participants were recruited three months to five years following their child ending treatment for cancer and were identified via their personal identification number (via the Swedish Childhood Cancer Registry and Swedish Tax Agency). Parents were invited via postal invitation letters and could opt-out via post, online, telephone, or e-mail. Those who did not opt-out or consent, within four weeks, received up to five telephone calls and/or one postal reminder.

Results: Of 509 invited 164 (32.2%) opted out, 78 (47.6%) via post, 53 (32.3%) via telephone, 24 (14.6%) online, and 6 (3.7%) via e-mail, 88 (53.7%) opted out after at least one telephone call and/or postal reminder. There was a trend for parents with lower educational levels to opt-out. No need of psychological support, lack of time, and no interest in internet-administered self-help were frequently reported reasons for non-participation in response to closed questions. Answers to an open question agreed with these findings and revealed additional reasons for non-participation, including study invitation timing.

Conclusions: Results emphasise the importance of using different opt-out modes and suggest future research should consider how to widen study participation for parents with lower education levels. Self-identifying a need for psychological support, study invitation timing, and the acceptability of internet-administered self-help are factors important for participation and should be considered in future research to increase recruitment rates.

Trial registration: ISRCTN57233429

For peer review only

For peer review only - http://bmjopen.bmj.com/site/about/guidelines.xhtml

STRENGTHS AND LIMITATIONS OF THIS STUDY

- Parents invited are a nationally representative sample of parents of children treated for cancer.
- The use of different modes of opt-out (e.g., post, online, telephone, or e-mail) has rarely been investigated.
- The use of closed and open questions allows for a broad understanding of reasons for non-participation which can inform strategies to increase recruitment rates to future clinical trials.
- The study was not designed to compare recruitment rates across different strategies and conclusions concerning the effectiveness of opt-out versus opt-in strategies cannot be drawn.
- We did not collect sociodemographic variables of non-responders and therefore it was not possible to examine whether the sample of parents who opted out, or chose to participate, are representative of the overall population in relation to key sociodemographic variables.

Key words

Cancer, Internet-administered CBT, Low-intensity CBT, Non-participation, Opt-out, Parents, Recruitment.

For peer review only

INTRODUCTION

Background

Difficulties with recruitment into clinical trials are common [1], significantly contributing to research waste.[2] Insufficient recruitment rates have been cited as the leading cause of clinical trials being terminated [3] and reviews have found only 56% of publically funded clinical trials reach their target sample size.[4] Difficulties with recruitment into clinical trials may result in delays to the generation of robust evidence to inform decisions to adopt interventions into routine healthcare.[5] Whilst there is an increased focus on identifying effective clinical trial recruitment strategies [6] current evidence is limited [7] with little high quality evidence to inform decisions concerning the use of different recruitment strategies in clinical trials.[6]

The ENGAGE study [8,9] is a single-arm feasibility trial of a guided, internet-administered low-intensity cognitive behavioural therapy (LI-CBT)-based intervention, designed to target common mental health difficulties (e.g., depression and generalised anxiety disorder) in parents of children treated for cancer.[10] The ENGAGE study examines clinical (e.g., intervention drop-out, adherence, and acceptability) and procedural (e.g., recruitment and retention) uncertainties. Examining recruitment uncertainties is important given the recruitment of participants into mental health intervention trials is particularly challenging.[11] Barriers may include a reluctance to seek help,[12] stigma,[13,14] and low perceived need for support.[15] Recruitment into clinical trials of e-mental health (e-MH) interventions (e.g. internet-administered CBT), has been identified as particularly challenging, [16] with barriers including lack of trust, poor digital health literacy, low confidence in using technology, low acceptability, and preferences for face-to-face interventions.[17-20]

Given challenges with recruitment into e-MH interventions trials, there is a need to examine the effectiveness of different recruitment strategies to increase recruitment rates. One strategy associated with increased recruitment rates and less biased samples is the opt-out strategy.[21] Potential participants invited into a study are provided with a method of actively declining contact with the research team (opting out) if they do not wish to participate in or receive additional study information. Those who do not opt out are contacted by the research team, for example via the telephone, to provide more study information. Contacting non-responders may result in increased recruitment rates in comparison to opt-in strategies, whereby potential participants must take action to signal willingness to participate to the research team.[1,7,11,22] An opt-out strategy may be particularly effective for e-MH intervention trials.[16] Mental health difficulties such as depression include lack of motivation, concentration difficulties, lack of belief in deserving intervention, and negative expectations concerning recovery, which have been found to be barriers to help-seeking.[16,23,24] An opt-out strategy may facilitate participation e.g., potential participants experiencing low levels of motivation or problems with self-initiating behaviour [25] are contacted by the research team and

For peer review only - http://bmjopen.bmj.com/site/about/guidelines.xhtml

provided with an opportunity to receive more information. Indeed, opt-out strategies been found to result in the recruitment of populations who are less healthy and more functionally impaired than opt-in strategies.[21] Whilst successfully used with adult and paediatric populations,[26] to the best of our knowledge, an opt-out strategy has not been used with parents of children treated for cancer.

Furthermore, literature is scarce concerning reasons for non-participation reported by parents of children treated for cancer. Wider literature suggests common reasons include lack of perceived personal benefit, time, and energy and preconceptions about research.[27,28] Whilst qualitative research with parents of children treated for cancer has been undertaken to inform the development of recruitment strategies for clinical trials,[29] to the best of our knowledge the population's reasons for non-participation have not been investigated. Understanding reasons for non-participation may enable the identification of modifiable barriers to participation (e.g., treatment preferences, burden of trial procedures) [14] and inform the development of recruitment strategies to help overcome these barriers.[14]

Aims and objectives

Primary objectives examined opt-out and consent rates, mode and time point of opt out, and sociodemographic characteristics of those who opted out versus chose to participate in the ENGAGE study.[8,9] Secondary objectives examined reasons for non-participation.

METHODS AND ANALYSIS

Study design

A cross-sectional survey with closed and open questions examining opt-out rates and reasons for non-participation nested within the ENGAGE study [8,9] reported, where applicable, in line with the Checklist for Reporting of Survey Studies (CROSS).[30]

Participants

Eligible participants were: (1) parents of a child diagnosed with cancer during childhood (0-18 years) who had completed cancer treatment three months to five years previously; (2) living in Sweden; (3) able to read and write Swedish; (4) able access to e-mail, the internet, and a mobile telephone and/or Bank ID; and (5) self-reporting a need for psychological support related to the child's cancer disease and treatment and a wish to work with the intervention. Parents were excluded if they: (1) had a self-reported or psychologist-assessed (based on the Mini-International Neuropsychiatric Interview, M.I.N.I.[31]) severe and enduring mental health difficulty; and/or misuse of alcohol, street drugs, and/or prescription medication; (2) were acutely suicidal; and/or (4) attended psychological treatment.

Recruitment

For peer review only - http://bmjopen.bmj.com/site/about/guidelines.xhtml

Potential participants were recruited using two strategies: (1) postal study invitation letters; and (2) online advertisements via patient organisations and interest groups. In this publication recruitment procedures and data associated with postal study invitation letters is reported.

Personal identification numbers of children who completed cancer treatment three months to five years previously were obtained from the Swedish Childhood Cancer Registry and linked to the names and addresses of their parent(s) via NAVET, a population registry held by the Swedish Tax Agency. Parents were invited in blocks of 100 until the target sample size (N=50) was reached. The first block invited was pre-selected with parents of children treated for cancer who ended treatment near to five years selected. The following four blocks were randomly selected. A de-identified list of all parents provided by NAVET was prepared by a research team member. To ensure allocation concealment a member of the U-CARE web-development team, external to the research team, used a computer generated simple randomization procedure to allocate parents into recruitment blocks. Postal study invitation letters were sent to parents' home addresses and included: (1) a study invitation letter, signed by the principle investigator (LvE) and a parent research partner (PRP); (2) a study information sheet and link to a secure website, the U-CARE-portal (Portal, an in-house platform which hosted the study and is designed to deliver internet-administered psychological interventions and support the collection of research data.[32,33]); (3) a reply slip to register potential interest; and (4) an opt-out form and reasons for non-participation survey; and (5) a freepost envelope. The ENGAGE study also included a Study Within A Trial (SWAT) investigating the effect of personalised versus non-personalised study invitation letters on recruitment and retention,[9] and results will be published in a separate publication.

Parents could access study information, in text and video format, and provide consent via the Portal. Parents who wished to opt out could do so by completing an opt-out form and reasons for non-participation survey via the Portal or by paper via the post (see Outcomes). Parents could also opt out by telephoning or e-mailing the research team.

Procedure

Opt-out and reminder telephone calls

Parents could sign an online form via the Portal using BankID, a secure electronic citizen authentication system used in Sweden, and were presented with a short reasons for non-participation survey (see Outcomes). Alternatively, parents could complete a paper-based survey, provide their name and ink signature, and return via the post. Parents who opted out via telephone or e-mail were asked to optionally provide a reason for non-participation.

For peer review only - http://bmjopen.bmj.com/site/about/guidelines.xhtml

Parents who did not respond (e.g., opt out or provide consent) within four weeks upon the first postal study invitation letter, received up-to five telephone reminder calls over four weeks. Telephone numbers were identified using internet search engines. A maximum of five reminder calls were made over four weeks, intended to confirm receipt of the postal study invitation and provide an opportunity to ask questions, or opt out from the study and further contact. Voicemails were left if the voicemail inbox had a personal recording clearly stating the parent's name. Communication between the parent and research team was documented in a recruitment case report form (CRF). If a telephone number could not be identified, a study invitation reminder letter and study invitation letter was resent. Personal details of parents who opted out were deleted from the research database.

Informed consent, screening, and baseline

Parents who chose to participate provided informed consent via the Portal and were contacted by the research team to schedule an eligibility interview with a licenced psychologist over the telephone. All parents were required to complete a baseline assessment via the Portal, or telephone if preferred. Full study procedures can be found in the ENGAGE study protocol.[8]

Intervention

The intervention is an internet-administered, guided, LI-CBT based intervention (the EJDeR intervention; intErnetbaserad sJälvhjälp för förälDrar till barn som avslutat en behandling mot canceR).[10] Following Phase I of the Medical Research Council's (MRC) complex interventions framework [35] development of EJDeR was informed by a systematic review,[35] qualitative interview studies,[36,37] a single-arm trial,[38] participatory action research,[39] and a cross-sectional online survey.[40] A Parent Research Partner (PRP) group of two mothers and two fathers of a child treated for cancer informed further refinements.[10] EJDeR is delivered on the Portal, and designed to be worked with over 12 weeks and consists of four modules: (1) Introduction and Psychoeducation; (2) Behavioural Activation; (3) Worry Management; and (4) Relapse Prevention.[10] Parents are guided by e-therapists, including an initial assessment session via a secure inbuilt video conferencing system or telephone call, weekly written messages via the Portal, and a "booster" session mid-way through the intervention, via video conferencing or telephone call.

Outcomes

An online and paper-based survey, consisting of 13 items, in Swedish. The survey comprises two subsections: (1) sociodemographic characteristics (4 items) and (2) reasons for non-participation (9 items). An open question at the end of the survey offered an opportunity for parents to provide any reasons for non-participation.

Sociodemographic characteristics

For peer review only - http://bmjopen.bmj.com/site/about/guidelines.xhtml

Background and sociodemographic characteristics was collected from parents who opted out via the online or paper-based survey: (1) gender (male/female/other); (2) age (years); (3a) relationship status (yes/no); (3b) cohabitation status (yes/no); and (4) highest level of education (free-text). Parents opting out via the telephone or e-mail were not asked to provide sociodemographic characteristics.

Reasons for non-participation

Parents were asked a closed, multiple choice question regarding reasons for non-participation, reasons were informed by previous research, [41,42] and parents could select all applicable reasons: (1) lack of time; (2) physical health; (3) not wanting to participate in research; (4) not liking to talk about personal problems; (5) too tired; (6) not interested in internet-administered self-help; (7) no need for psychological help; (8) child's health condition; and (9) child has recently passed away. The open question at the end of the survey was: "If you do not want to participate in the project for reasons other than those mentioned above, please write these below".

Whereby the parent opted out via telephone or e-mail, a CRF was completed by the research team. The CRF included the reason(s) for non-participation listed in the survey and a free-text box in response to the open question.

Sample size

No formal sample size calculation was made [43] however recommendations for assessing feasibility outcomes were followed [44] with an aim to recruit 50 parents. It was estimated approximately 600 parents would be invited to meet the sample size.[9]

Progression criteria

A priori progression criteria [45] were set, these can be found in the published protocol for the ENGAGE study.[8] A criteria that ≥9% of the total population invited into the study via the Swedish Childhood Cancer Registry chose to participate was set.

Data analysis

Data analysis was performed in Excel and SPSS for Windows, version 27.0 (IBM Corporation). Recruitment flow via postal study invitation letters is illustrated using an adapted CONSORT flow diagram.[46] Descriptive statistics were used to describe numbers and proportions who; opted out; opted out via each mode (postal/online/telephone/e-mail) and at each time-point (before/after telephone and/or postal reminder/s); and responded to a multiple choice question regarding reasons for non-participation. Study sample sociodemographic characteristics are described using descriptive statistics. Chi-square test was used to examine differences regarding categorical variables, and t-tests were calculated for continuous variables.

For peer review only - http://bmjopen.bmj.com/site/about/guidelines.xhtml

An inductive qualitative content analysis [47] managed using Microsoft Excel and Word was adopted to analyse responses to the open question in the reasons for non-participation survey. Free-text responses were read by two authors (JH and AvE) to gain an overall understanding of content. Next, condensed meaning units were identified (e.g., text sharing a common meaning) and assigned a code by JH and AvE separately. JH reviewed initial meaning units and codes to identify any differences in coding, with differences discussed by JH, AvE, and a third author (JW) if required. Codes were sorted into categories and subcategories by JH and AvE separately. As categories were identified, some content was recognised as not relating to reasons for non-participation, but included important content not to be discarded, for example, gratitude for the research. Categories were subsequently classified into two themes: Reasons for non-participation and Content not related to reasons for non-participation. JH, AvE, and JW met regularly to discuss categories and subcategories and ensure content was mutually exclusive. All responses were subsequently re-read by JH to check agreement with identified categories and subcategories. JH and AvE jointly prepared brief descriptions for categories and subcategories. Trustworthiness was established using peer examination of a supervising researcher (JW), two coders (JH and AvE) working independently, and record keeping.[48]

Other reasons for non-participation provided by parents via telephone or e-mail to the research team and recorded on the recruitment CRF were also categorised by JH and AvE separately, with all categorisations discussed with JW to obtain consensus.

Patient and Public involvement

The PRP group (two mothers and two fathers) provided feedback on the design and wording of the study invitation letter. PRPs were sent a study invitation letter draft via e-mail and asked to comment on wording, content, format, and design, and whether to include a PRP's name and signature on the letter alongside that of the principle investigator (LvE). Parents did not suggest any changes and considered the letter to be concise, validating, and informative and agreed the name and signature of a PRP should be included.[9] Patient and public involvement may have been improved by engaging PRPs in drafting the opt-out survey, for example informing the options available in the multiple choice question regarding reasons for non-participation.

RESULTS

Opt-out and consent rates

Recruitment via postal study invitation letters into the ENGAGE feasibility study took place between 3rd July 2020 and 30th November 2020. 509 parents were identified and invited. 32.2% (164/509) opted out, and 11.8% (60/509) provided consent, exceeding the a priori recruitment rate progression

For peer review only - http://bmjopen.bmj.com/site/about/guidelines.xhtml

criteria of ≥9% invited via the Swedish Childhood Cancer Registry.[8,45]. 11.0% (56/509) were found to be eligible for the study.

Three parents were initially registered as opting out via the telephone by a partner who had also received a study invitation letter. Subsequently, the opt-out protocol was adapted and opting out via a partner was no longer an option. Recruitment flow via postal study invitation letters can be seen in Figure 1.

-INSERT FIGURE 1 ABOUT HERE-

For peer review only

Mode of opting-out

The most frequent mode of opt-out was via the post (78/164, 47.6 %), followed by telephone (53/164, 32.3%), the Portal (24/164, 14.6%), and e-mail (6/164, 3.7%). Three parents (3/164; 1.8%) opted out via their partner over the telephone. Two who initially opted out via telephone, subsequently opted out via the post, resulting in a total of n=80 opt-outs via the post.

Time point of opting-out

The majority of opt-outs occurred after one or more telephone reminder call and/or postal reminder (88/164, 53.7%). The mean number of days between a postal study invitation letter being sent and an opt-out was 30.0 days (SD=20.6, range = 2-158).

Sociodemographic characteristics of parents who opted out versus choose to participate

Sociodemographic characteristics of parents (n=105) who opted out and provided sociodemographic data and parents (n=56) who consented and were screened for eligibility is shown in Table 1. No differences between groups were found, though there was a trend for parents with lower educational levels to opt out, and parents with a tertiary education to choose to participate.

Table 1. Sociodemographic characteristics for parents who opted out and who consented and were screened for eligibility

Characteristic	Opted out (n=105)[a]	Consented and screened for eligibility (n=56)	t value, χ2 value	p Value
**Age, mean (SD, range)**	44.8 (7.6, 29-68)	43.5 (7.2, 32-62)	-0.988	0.325
Missing (n, %)	19 (18.1)	0 (0)		
**Gender (n, %)**				
Female	54 (51.4)	33 (58.9)	0.001	0.978
Male	38 (36.2)	23 (41.1)		

For peer review only - http://bmjopen.bmj.com/site/about/guidelines.xhtml

Missing	13 (12.4)	0 (0)		
**Partner (n, %)**				
Yes	86 (81.9)	47 (83.9)	1.996	0.158
No	8 (7.6)	9 (16.1)		
Missing	11 (10.5)	0 (0)		
If yes, cohabiting				
Yes	78 (90.7)	46 (97.9)	1.035	0.309
No	5 (5.8)	1 (2.1)		
Missing	3 (3.5)	0 (0)		
**Level of education (n, %)[b]**				
Lower secondary	6 (5.7)	1 (1.8)	7.629	0.178
Upper secondary	30 (28.6)	11 (19.6)		
Post-secondary non-tertiary	5 (4.8)	3 (5.4)		
Tertiary education	50 (47.6)	41 (73.2)		
PhD	2 (1.9)	0 (0)		
Not possible to categorize	1 (1.0)	0 (0)		
Missing	11 (10.5)	0 (0)		

[a]105 parents who opted out provided some sociodemographic data. Sociodemographic characteristics were collected from parents who opted out by paper via the post (n=80) or Portal (n=24). One parent opted out via e-mail and voluntarily provided some sociodemographic data.

[b]Level of education was self-reported as a free-text response, with responses categorised by research team.

Reasons for non-participation

Closed question

83.5% (137/164) responded to the closed question about reasons for non-participation. The most frequently reported reason was not experiencing any need for psychological support (93/137, 67.9%) followed by not having enough time (53/137, 38.7%), and not interested in using internet-administered self-help (23/137, 16.8%). Responses are shown in Table 2 and presented separately for female and male gender where data was available.

Table 2. Reasons for non-participation in response to the closed multiple-choice question

Reasons	Female (n=50[a])		Male (n=35[a])		Total (n=137[b])	
	n	%	n	%	n	%

For peer review only - http://bmjopen.bmj.com/site/about/guidelines.xhtml

I do not experience any need for psychological support	32	64.0	27	77.1	93	67.9
I do not have enough time	24	48.0	19	54.3	53	38.7
I am not interested in using internet-administered self-help	10	20.0	8	22.9	23	16.8
I am too tired to participate in a research project	9	18.0	6	17.1	20	14.6
I do not want to participate in a research project	7	14.0	8	22.9	17	12.4
I do not like talking about my personal problems	5	10.0	4	11.4	11	8.0
My physical health is hindering me	3	6.0	1	2.9	4	2.9
I do not want to participate because of my child's health condition	2	4.0	0	0	2	1.5
I do not want to participate because my child recently died	1	2.0	0	0	1	0.7

[a]n=85 parents provided data regarding gender (female/male)

Open question

Thirty parents responded to the open question regarding reasons for non-participation in the paper-based or online survey. 21 provided a response via telephone or e-mail recorded in the CRF. Content analysis of responses within the theme Reasons for non-participation resulted in seven categories: Timing, Emotional wellbeing, Support, Perceived ineligibility, Other commitments, and Reluctance to engage in internet-administered self-help. Responses within the theme Content not related to reasons for non-participation were categorised as: Not for me, but…. and When my child was ill. Themes, categories, subcategories, and quotations are shown in Table 3.

Table 3. Themes, categories, subcategories, and quotations. Categories are presented in order of number of included responses whereas subcategories are presented alphabetically

Theme	Category	N (n=51)	Subcategory	Quotation (P: reported by parent; R: recorded by researcher)
Reasons for non-participation	Timing	20	Earlier need	"We would have needed help a long time ago, but we have managed well and right now we are doing well" (P)<br>"The need was bigger right after completed treatment" (R)
			Not right now	"Could be interesting another time" (P)

For peer review only

For peer review only - http://bmjopen.bmj.com/site/about/guidelines.xhtml

	Emotional wellbeing	16	Coping strategies	"We/I don't have the energy to be involved in sickness anymore, more than I am" (P)<br>"Sure, it was heavy and tough, but life is what you make it" (P)
			Family wellbeing	"… my son is well today, and us parents as well" (P)<br>"Child is doing fine" (R)
			Fatigue	"We are tired and it's always a lot, but it's not related to the cancer [the child's]" (P)
			No need	"Feeling no need for help, feeling good" (P)<br>"Felt no need for therapy" (R)
	Support	13	Professional support	"I have processed a lot on my 'own' with the help from a psychologist" (P)<br>"Has received psychological treatment" (R)
			Social support	"[I've come a long way] with help from […] and close ones" (P)<br>"Had a good social network helping the family" (R)
	Perceived ineligibility	11	N/A	"Cannot read Swedish" (P)<br>"In psychological treatment" (R)<br>"I'm too broken in many ways, and unfortunately I cannot participate" (P)<br>"Did not feel suitable for the project" (R)
	Other commitments	7	N/A	"Unfortunately I don't have the time to participate" (P)<br>"Too busy to participate" (R)
	Reluctance to engage in internet-administered self-help	2	N/A	"Did not feel comfortable with the internet" (R)
Content not related to	Not for me, but …	19	Gratitude	"Great that you are doing this" (P)<br>"Very positive about project" (R)

For peer review only - http://bmjopen.bmj.com/site/about/guidelines.xhtml

reasons for non-participation			Need among others	"I hope many participate, there is really a need for support after treatment" (P)<br>"Would love to help other parents" (R)
	When my child was ill	10	Available care	"The care after completing treatment was non-existent" (R)
			Cancer illness	"My son was critically ill when the intestine broke" (P)
			Family life	"When [child] was diagnosed with cancer, my husband and I tried to keep things as 'normal' as possible" (P)

Reasons for non-participation

The category Timing includes the subcategories: *Earlier need* and *Not right now*. *Earlier need* concerns the invitation coming "too late" i.e. after treatment completion, with parents positing hypothetical participation had they been approached earlier. *Not right now* concerns not currently interested in participation, however potential interest another time.

The category Emotional wellbeing includes the subcategories: *Coping strategies*, *Family wellbeing, Fatigue,* and *No need*. *Coping strategies* concerns use of coping strategies. Parents stated wanting to forget "what has been" (e.g., the cancer experience) and a reluctance to "go back and think about these things" or to remain involved with sickness. Conversely, parents reported focusing on the positive, accepting their situation, holding a "positive" attitude towards difficult situations, and post-traumatic growth. Parents could report distancing from the cancer experience in the past and present as well as a want to "focus on the positive" in the future. *Family wellbeing* concerns the entire family, for example as their child was in good health, psychological support for the parent was not considered needed. *Fatigue* concerns being too tired to participate and *No need* concerns parents feeling well and not experiencing a need for psychological support.

The category Prior support includes the subcategories: *Professional support* and *Social support*. *Professional support* concerns receipt of current or past support from a health professional, for example, a psychologist or counsellor whereas *Social support* concerns support and help by family, friends, and pets.

The category Perceived ineligibility concerns parents not considering themselves eligible for the study, e.g. not fulfilling the inclusion criteria for example insufficient knowledge of Swedish, undergoing psychological treatment as well as other reasons such as being "too broken" to participate.

For peer review only

For peer review only - http://bmjopen.bmj.com/site/about/guidelines.xhtml

The category Other Commitments concerns life commitments occupying parents' time, reference was also made to too many invitations regarding participation in research and a wish to have free time.

The category Reluctance to engage in an internet-administered intervention included responses whereby parents reported being uncomfortable with the internet or preferring face-to-face treatment.

Content not related to reasons for non-participation

The category Not for me, but… includes the subcategories: *Gratitude* and *Need among others*. *Gratitude* concerns the importance of the study and gratitude that it was carried out. *Need among others* concerns a hope that other parents would participate and receive help, and that there is a need for support for parents after their child's cancer treatment.

The category When my child was ill includes the subcategories: *Available care*, *Cancer illness,* and *Family life*. *Available care* concerns the care during the child's illness and treatment. Parents mentioned there had been little care available for their wellbeing after their child had completed treatment. *Cancer illness* concerns details about the child's illness and treatment, for example the duration of the treatment or the child having been ill since birth. *Family life* concerns experiences during their child's illness and treatment, for example, striving for normality or difficult family events such as separating from a partner.

DISCUSSION

Summarising the main findings, the opt-out rate was 32.2%, the consent rate via postal study invitation letters was 11.8%, and 11.0% were eligible for the study. The most frequent mode of opt-out was by paper via the post (47.6 %). The majority (53.7%) opted out of further contact with the research team after one or more reminder via telephone and/or post. Although not significant, there was a trend for parents with lower educational levels to opt out, and parents with a tertiary education choosing to participate. Common reasons for participation in response to the closed question were not experiencing any need for psychological support not having enough time, and not having an interest in using internet-administered self-help. Content analysis of responses to the open question agreed with results from the closed question, with reasons for non-participation including emotional wellbeing, receipt of psychological support, and commitments limiting time to participate. Additional reasons were suggested, including study invitation timing, and parents not perceiving themselves as eligible for the study.

The overall consent rate exceeded the recruitment target outlined in study progression criteria.[8,45] Use of an opt-out strategy has demonstrated higher recruitment rates when compared to opt-in strategies.[7,49,50] For example, randomised controlled trials (RCTs) comparing opt-out versus opt-in

For peer review only - http://bmjopen.bmj.com/site/about/guidelines.xhtml

strategies have found recruitment rates of 40-50% for opt-out, versus 26-38% for opt-in.[21,51]. However, studies utilising opt-out strategies are highly heterogeneous, with variations in recruitment rates, populations, designs, and interventions [49] and few RCTs have compared opt-out versus opt-in strategies [7,50], limiting conclusions regarding effectiveness. The ENGAGE study did not include an opt-in comparison arm, making it impossible to determine whether successful recruitment was a result of an opt-out strategy.

Study recruitment took place during the COVID-19 pandemic. Given physical distancing recommendations in Sweden, as in many countries globally,[52] people have spent increased time at home. Research has observed increased parental stress associated with physical distancing measures and the closure of schools and childcare facilities.[53] A retrospective online survey conducted in the United States found that 71.1% of parents reported increases in parenting-specific stress from before COVID-19.[53] Further, a survey conducted in the United Kingdom indicated 85% of parents of children with cancer were worried about the virus, for example risk of infection, reduced healthcare provision, and increased risk of social isolation, and 70% no longer deemed the hospital a safe place.[54] The context of a pandemic may have facilitated recruitment, e.g., parents may have been experiencing elevated levels of fear and anxiety. Conversely, the pandemic may have been a barrier to participation for some, given parents of children with cancer commonly focus on their child's needs, at the expense of their own wellbeing.[35-37]

The overall opt-out rate in the present study is higher than a 19% opt-out rate found in a longitudinal cohort pilot study of angina patients [21] and a 21% opt-out rate seen in a survey study regarding end of life care.[51] However, existing literature on opt-out strategies tends to be on observational studies, as opposed to intervention or health service-orientated research [49] making it difficult to compare results with the wider literature. When comparing the opt-out rate in the present study to non-participation rates in intervention studies for parents or informal caregivers using opt-in strategies, findings are also varied. In an RCT of an intervention for parents of children with cancer to reduce exposure to tobacco smoke, 24% approached about study participation within a clinic setting declined participation [55] and in a pilot RCT of guided CBT self-help for informal caregivers of stroke survivors, 15% invited by post actively declined participation.[56] Further, in an RCT of a psychological intervention for cancer patients in palliative care and their family caregivers 65.9% of potential participants actively declined participation.[57]

Interestingly, the most frequent mode of opt-out was paper via the post. In comparison with the general adult population, parents of a child treated for cancer three months to five years following end of treatment may be assumed to be younger, with good digital knowledge and skills.[58] Therefore it may have been anticipated that parents would prefer opt-out online. Systematic reviews and meta-

For peer review only - http://bmjopen.bmj.com/site/about/guidelines.xhtml

analyses have however shown postal survey response to be, in general, higher than online surveys.[59,60] One potential reason is rising concerns about security and privacy in the digital age.[61,62] Indeed, common barriers to engaging in internet-administered interventions include security and confidentiality concerns [19] Possibly, parents may have experienced opt-out via the post as implying higher levels of security and privacy than using an electronic personal identification method (BankID) to opt out online. However, variation in response rate across the four opt-out modes suggests it is important to offer potential participants multiple modes of opt-out.

The trend for parents with an upper secondary school education to opt out of the study while parents with a tertiary education chose to participate, is supported by the wider literature. For example, parents of adolescents with psychosocial difficulties who have lower levels of education perceive more barriers towards accessing psychosocial care, than parents with a university level education.[63] Moreover, evidence suggests online survey responders tend to be more highly educated than non-responders [64] and adults with lower levels of education struggle to find health information online [65,66] and require more guidance to use internet-administered interventions.[66] Higher education levels are also associated with increased effectiveness and higher completion rates of guided internet-administered CBT interventions in a general adult population.[67] Future research is needed to improve the acceptability and feasibility of internet-administered interventions for people with lower education levels.

In line with previous research with depressed adult populations,[68,69] the most common reason for non-participation was not perceiving a need for psychological support. Similar findings were found in psychological intervention studies for adult cancer patient/caregiver dyads [3] and informal caregivers of stroke survivors.[56] Around 14-30% of parents of children treated for cancer report clinically relevant levels of mental health difficulties, such as depression, anxiety, and posttraumatic stress.[31,70,71] Therefore at a group level, a majority of parents report levels of psychological distress within a normal range [35] and it can be anticipated most do not experience a need for psychological support. However, as the 11.8% recruitment rate is under the prevalence rate of psychological distress in the population, some parents in need of psychological support did not consent and research to increase participation among this group is warranted.

Study invitation timing was the most commonly cited reason for non-participation in response to the open question, corroborating findings of a similar study conducted with parents of children with cancer examining potential barriers to recruitment.[29] Parents found it difficult to identify the most appropriate time point for intervention, as periods of increased distress may differ between parents.[29] Other research has found invitation timing plays a significant role in a person's decision to participate in studies of psychosocial interventions for couples coping with cancer [72] and that it is

For peer review only - http://bmjopen.bmj.com/site/about/guidelines.xhtml

difficult to find an optimal time point to recruit cancer patient/caregiver dyads into intervention research.[73] A potential solution may be to not place any time limit from diagnosis/ end of treatment.

Finally, lack of interest in internet-administered self-help was a common reason for non-participation. Despite well demonstrated clinical effectiveness of e-MH interventions[74], difficulties with recruitment into trials are common [75] and rates of implementation are low.[76] Barriers include lack of trust, poor digital health literacy, low levels of confidence in using digital technology, and preferences for face-to-face interventions.[17-20] Research indicates acceptability may increase when potential users are provided with information about intervention content and/or effectiveness [18,77] and future research may wish to examine the provision of additional intervention information, including for example screenshots or videos, on recruitment rates.

Ethical considerations

The study was approved by the Regional Ethical Review Board in Uppsala, Sweden (Dnr: 2017/527) and was conducted in accordance with the Helsinki Declaration and Good Clinical Practice (GCP) guidelines.

One concern around an opt-out strategy is the use of repeated reminders/contact attempts, and potentially coercing participants.[21] While research ethics committees often require researchers to utilise opt-in recruitment strategies to protect participant confidentiality and (medical) privacy [78], this minimises the opportunity for researchers to speak with potential participants directly to explain the study.[79] Consistent with other research utilising opt-out recruitment whereby few concerns have been raised,[78] only one complaint concerning reminder calls was received during the study, indicating an opt-out recruitment strategy is acceptable to the population.

Strengths and limitations

This study has several strengths. First, parents were identified and randomly invited into the study via the Swedish Childhood Cancer Registry and a population registry held by the Swedish Tax Agency (NAVET). As such, invited parents are a nationally representative sample of parents of children treated for cancer in Sweden. Second, parents were provided with different modes of opt-out (e.g., post, online, telephone, or e-mail). To the best of our knowledge, this is the first study to compare opt-out rates across different opt-out modalities. Third, by using both closed and open questions to examine reasons for non-participation, results provide us with a broad understanding of reasons for non-participation which may inform the development of future recruitment strategies. The study also has limitations. First, it was not designed to compare recruitment rates across different strategies and conclusions on the effectiveness of the opt-out strategy versus other recruitment strategies cannot be drawn. Second, we did not collect sociodemographic variables of non-responders and therefore it was

For peer review only - http://bmjopen.bmj.com/site/about/guidelines.xhtml

not possible to assess whether the sample is representative of the overall population in relation to key sociodemographic variables. Some sociodemographic data (e.g., sex, age, geographical location) could have been obtained for all potential participants via the NAVET registry, which would have enabled comparisons between participants who consented, opted out, and were non-responders [80] however we did not include the collection of this data in the ethics application.

CONCLUSIONS

This is the first study to examine the use of an opt-out strategy within a psychological intervention study for parents of children treated for cancer. Though recruitment into clinical trials is challenging, projected recruitment rates were exceeded. Developing a more comprehensive understanding of reasons for non-participation, may help inform planning and decision-making concerning study design, and potentially enhance recruitment rates and reduce research waste in future trials of internet-administered interventions for both parent/caregiver and wider populations. Future research may wish to compare opt-out versus opt-in strategies within an RCT and embed qualitative interviews to explore reasons for non-participation and develop a more in-depth understanding of potential barriers to trial participation.

ACKNOWLEDGEMENTS

The authors thank the four PRPs (Helena Börjesson, Martin Hedqvist, Anna Mautner, and Erik Olsson) for their feedback on study invitation material. In addition, we wish to thank Fabian Holmberg, Ian Horne, and Ylva Hägg Sylvén (Portal team members) for their assistance with developing the opt-out procedure on the Portal.

AUTHORS' CONTRIBUTIONS

Author contributions are written in accordance with the CRediT statement: JH: formal analysis, investigation, data curation, writing - original draft, visualization; JW: methodology, formal analysis, writing – original draft, supervision, project administration; AvE: validation, investigation, data curation, formal analysis, writing – review and editing; PL: resources, writing – review and editing; LvE: conceptualization, methodology, resources, writing – review and editing, supervision, project administration, funding acquisition.

FUNDING

This work is supported by the Swedish Research Council (grant number K2015-99X-20836-08-4 and 2018-02578), the Swedish Cancer Society (grant number 150673 and 180589), the Swedish Childhood Cancer Foundation (grant number PR2017-0005), and funding via the Swedish Research Council to

For peer review only - http://bmjopen.bmj.com/site/about/guidelines.xhtml

U-CARE, a Strategic Research environment (Dnr 2009–1093). The funders had no role in study design, data collection and analysis, decision to publish, or preparation of the manuscript.

COMPETING INTERESTS

The authors have no competing interests to declare

ABBREVIATIONS

CBT: Cognitive Behavioural Therapy
CRF: Case Report Form
e-MH: e-Mental Health
LI-CBT: Low-Intensity Cognitive Behavioural Therapy
MRC: Medical Research Council

FIGURES

Figure 1. Recruitment flow via postal study invitation letters

REFERENCES

1. Bower P, Brueton V, Gamble C, et al. Interventions to improve recruitment and retention in clinical trials: a survey and workshop to assess current practice and future priorities. *Trials* 2014;15:399. doi:10.1186/1745-6215-15-399.
2. Moher D, Glasziou P, Chalmers I, et al. Increasing value and reducing waste in biomedical research: who's listening? *Lancet* 2016;387(10027):1573-1586. doi:10.1016/S0140-6736(15)00307-4.
3. Williams RJ, Tse T, DiPiazza K, et al. Terminated trials in the ClinicalTrials.gov results database: evaluation of availability of primary outcome data and reasons for termination. *PLoS One* 2015;10(5):e0127242. doi:10.1371/journal.pone.0127242.
4. Walters SJ, Bonacho Dos Anjos Henriques-Cadby I, Bortolami O, et al. Recruitment and retention of participants in randomised controlled trials: a review of trials funded and published by the United Kingdom Health Technology Assessment Programme. *BMJ Open* 2017;7(3):e015276. doi:10.1136/bmjopen-2016-015276.
5. Richards DA, Ross S, Robens S, et al. The DiReCT study - improving recruitment into clinical trials: a mixed methods study investigating the ethical acceptability, feasibility and recruitment yield of the cohort multiple randomised controlled trials design. *Trials* 2014;15:398. doi:10.1186/1745-6215-15-398.
6. Treweek S, Bevan S, Bower P, et al. Trial Forge Guidance 1: what is a Study Within A Trial (SWAT)? *Trials* 2018;19(1):139. doi:10.1186/s13063-018-2535-5.

For peer review only - http://bmjopen.bmj.com/site/about/guidelines.xhtml

For peer review only

7. Treweek S, Pitkethly M, Cook J, et al. Strategies to improve recruitment to randomised trials. *Cochrane Database Syst Rev* 2018;2(2):MR000013. doi:10.1002/14651858.MR000013.pub6.

8. Woodford J, Wikman A, Cernvall M, et al. Study protocol for a feasibility study of an internet-administered, guided, CBT-based, self-help intervention (ENGAGE) for parents of children previously treated for cancer. *BMJ Open* 2018;8(6):e023708. doi:10.1136/bmjopen-2018-023708.

9. Woodford J, Norbäck K, Hagström J, et al. Study within a trial (SWAT) protocol. Investigating the effect of personalised versus non-personalised study invitations on recruitment: An embedded randomised controlled recruitment trial. *Contemp Clin Trials Commun* 2020;18:100572. doi:10.1016/j.conctc.2020.100572.

10. Woodford J, Farrand P, Hagström J, et al. Internet-administered cognitive behavioral therapy for common mental health difficulties in parents of children treated for cancer: intervention development and description study. *JMIR Form Res* 2021;5(7):e22709. doi:10.2196/22709.

11. Liu Y, Pencheon E, Hunter RM, et al. Recruitment and retention strategies in mental health trials - a systematic review. *PLoS One* 2018;13(8):e0203127. doi:10.1371/journal.pone.0203127.

12. Mackenzie CS, Erickson J, Deane FP, et al. Changes in attitudes toward seeking mental health services: a 40-year cross-temporal meta-analysis. *Clin Psychol Rev* 2014;34(2):99-106. doi:10.1016/j.cpr.2013.12.001.

13. Clement S, Schauman O, Graham T, et al. What is the impact of mental health-related stigma on help-seeking? A systematic review of quantitative and qualitative studies. *Psychol Med* 2015;45(1):11-27. doi:10.1017/S0033291714000129.

14. Hughes-Morley A, Young B, Waheed W, et al. Factors affecting recruitment into depression trials: Systematic review, meta-synthesis and conceptual framework. *J Affect Disord* 2015;172:274-90. doi:10.1016/j.jad.2014.10.005.

15. Mojtabai R, Olfson M, Sampson NA, et al. Barriers to mental health treatment: results from the National Comorbidity Survey Replication. *Psychol Med* 2011;41(8):1751-61. doi:10.1017/S0033291710002291.

16. Woodford J, Farrand P, Bessant M, et al. Recruitment into a guided internet based CBT (iCBT) intervention for depression: lesson learnt from the failure of a prevalence recruitment strategy. *Contemp Clin Trials* 2011;32(5):641-8. doi:10.1016/j.cct.2011.04.013.

17. Apolinário-Hagen J, Kemper J, Stürmer C. Public acceptability of e-mental health treatment services for psychological problems: a scoping review. *JMIR Ment Health* 2017;4(2):e10. doi: 10.2196/mental.6186.

18. March S, Day J, Ritchie G, et al. Attitudes toward e-mental health services in a community sample of adults: online survey. *J Med Internet Res* 2018;20(2):e59. doi:10.2196/jmir.9109.

For peer review only

For peer review only - http://bmjopen.bmj.com/site/about/guidelines.xhtml

19. Schreiweis B, Pobiruchin M, Strotbaum V, et al. Barriers and facilitators to the implementation of eHealth services: systematic literature analysis. *J Med Internet Res* 2019;21(11):e14197. doi:10.2196/14197.

20. Vis C, Mol M, Kleiboer A, et al. Improving implementation of eMental Health for mood disorders in routine practice: systematic review of barriers and facilitating factors. *JMIR Ment Health* 2018;5(1):e20. doi:10.2196/mental.9769.

21. Junghans C, Feder G, Hemingway H, et al. Recruiting patients to medical research: double blind randomised trial of "opt-in" versus "opt-out" strategies. *BMJ* 2005;331(7522):940. doi:10.1136/bmj.38583.625613.AE.

22. Trevena L, Irwig L, Barratt A. Impact of privacy legislation on the number and characteristics of people who are recruited for research: a randomised controlled trial. *J Med Ethics* 2006;32(8):473-7. doi:10.1136/jme.2004.011320.

23. Roberts T, Miguel Esponda G, Krupchanka D, et al. Factors associated with health service utilisation for common mental disorders: a systematic review. *BMC Psychiatry* 2018;18(1):262. doi:10.1186/s12888-018-1837-1.

24. Shi W, Shen Z, Wang S, et al. Barriers to professional mental health help-seeking among Chinese adults: a systematic review. *Front Psychiatry* 2020;11:442. doi:10.3389/fpsyt.2020.00442.

25. Katon WJ. Epidemiology and treatment of depression in patients with chronic medical illness. Dialogues *Clin Neurosci* 2011;13(1):7-23. doi:10.31887/DCNS.2011.13.1/wkaton.

26. McCullough MB, Janicke D, Odar Stough C, et al. Barriers to recruitment in pediatric obesity trials: comparing opt-in and opt-out recruitment approaches. *J Pediatr Psychol* 2017;42(2):174-185. doi:10.1093/jpepsy/jsw054.

27. Nalubega S, Evans C. Participant views and experiences of participating in HIV research in sub-Saharan Africa: a qualitative systematic review. *JBI Database System Rev Implement Rep* 2015;13(5):330-420. doi:10.11124/jbisrir-2015-2051.

28. Rivers D, August EM, Sehovic I, et al. A systematic review of the factors influencing African Americans' participation in cancer clinical trials. *Contemp Clin Trials* 2013;35(2):13-32. doi:10.1016/j.cct.2013.03.007.

29. Canter KS, Vega G, Ramirez AP, et al. Strategies for successful recruitment and retention of parents in pediatric psychosocial eHealth interventions: a qualitative study in pediatric oncology. *J Pediatr Psychol* 2020;45(5):530-539. doi:10.1093/jpepsy/jsaa013.

30. Sharma A, Minh Duc NT, Luu Lam Thang T, et al. A Consensus-Based Checklist for Reporting of Survey Studies (CROSS). *J Gen Intern Med* 2021. doi:10.1007/s11606-021-06737-1.

31. Sheehan DV, Lecrubier Y, Sheehan KH, et al. The Mini-International Neuropsychiatric Interview (M.I.N.I.): the development and validation of a structured diagnostic psychiatric interview for DSM-IV and ICD-10. *J Clin Psychiatry* 1998;59 Suppl 20:22-33.

For peer review only - http://bmjopen.bmj.com/site/about/guidelines.xhtml

32. Grönqvist H, Olsson EMG, Johansson B, et al. Fifteen challenges in establishing a multidisciplinary research program on eHealth research in a university setting: a case study. *J Med Internet Res* 2017;19(5):e173. doi:10.2196/jmir.7310.
33. Sjöström J, von Essen L, Grönqvist H. The origin and impact of ideals in eHealth research: experiences from the U-CARE research environment. *JMIR Res Protoc* 2014;3(2):e28. doi:10.2196/resprot.3202.
34. Craig P, Dieppe P, Macintyre S, et al. Developing and evaluating complex interventions: the new Medical Research Council guidance. BMJ 2008;337:a1655. doi:10.1136/bmj.a1655.
35. Ljungman L, Cernvall M, Grönqvist H, et al. Long-term positive and negative psychological late effects for parents of childhood cancer survivors: a systematic review. *PLoS One* 2014;9(7):e103340. doi:10.1371/journal.pone.0103340.
36. Carlsson T, Kukkola L, Ljungman L, et al. Psychological distress in parents of children treated for cancer: an explorative study. *PLoS One* 2019;14(6):e0218860. doi:10.1371/journal.pone.0218860.
37. Ljungman L, Boger M, Ander M, et al. Impressions that last: particularly negative and positive experiences reported by parents five years after the end of a child's successful cancer treatment or death. *PLoS One* 2016;11(6):e0157076. doi:10.1371/journal.pone.0157076.
38. Ljungman L, Cernvall M, Ghaderi A, et al. An open trial of individualized face-to-face cognitive behavior therapy for psychological distress in parents of children after end of treatment for childhood cancer including a cognitive behavioral conceptualization. *PeerJ* 2018;6:e4570. doi:10.7717/peerj.4570.
39. Wikman A, Kukkola L, Börjesson H, et al. Development of an internet-administered cognitive behavior therapy program (ENGAGE) for parents of children previously treated for cancer: Participatory action research approach. *J Med Internet Res* 2018;20(4):e133. doi:10.2196/jmir.9457.
40. Woodford J, Wikman A, Einhorn K, et al. Attitudes and preferences toward a hypothetical trial of an internet-administered psychological intervention for parents of children treated for cancer: web-based survey. *JMIR Ment Health* 2018;5(4):e10085. doi:10.2196/10085.
41. Barnes M, Wiles N, Morrison J, et al. Exploring patients' reasons for declining contact in a cognitive behavioural therapy randomised controlled trial in primary care. *Br J Gen Pract* 2012;62(598):e371-7. doi:10.3399/bjgp12X641492.
42. Farrand P, Woodford J, Llewellyn D, et al. Behavioural activation written self-help to improve mood, wellbeing and quality of life in people with dementia supported by informal carers (PROMOTE): a study protocol for a single-arm feasibility study. *Pilot Feasibility Stud* 2016;2:42. doi:10.1186/s40814-016-0083-x.
43. Sim J. Should treatment effects be estimated in pilot and feasibility studies? *Pilot Feasibility Stud* 2019;5:107. doi:10.1186/s40814-019-0493-7.

For peer review only - http://bmjopen.bmj.com/site/about/guidelines.xhtml

44. Sim J, Lewis M. The size of a pilot study for a clinical trial should be calculated in relation to considerations of precision and efficiency. *J Clin Epidemiol* 2012;65(3):301-8. doi:10.1016/j.jclinepi.2011.07.011.

45. Mbuagbaw L, Kosa SD, Lawson DO, et al. The reporting of progression criteria in protocols of pilot trials designed to assess the feasibility of main trials is insufficient: a meta-epidemiological study. *Pilot Feasibility Stud* 2019;5:120. doi:10.1186/s40814-019-0500-z.

46. Schulz KF, Altman DG, Moher D; et al. CONSORT 2010 statement: updated guidelines for reporting parallel group randomized trials. *Ann Intern Med* 2010;152(11):726-32. doi:10.7326/0003-4819-152-11-201006010-00232.

47. Elo S, Kyngäs H. The qualitative content analysis process. *J Adv Nurs* 2008;62(1):107-15. doi:10.1111/j.1365-2648.2007.04569.x.

48. Krefting L. Rigor in qualitative research: the assessment of trustworthiness. *Am J Occup Ther* 1991;45(3):214-22. doi:10.5014/ajot.45.3.214.

49. Miller CJ, Burgess JF Jr, Fischer EP, et al. Practical application of opt-out recruitment methods in two health services research studies. *BMC Med Res Methodol* 2017;17(1):57. doi:10.1186/s12874-017-0333-5.

50. Treweek S, Pitkethly M, Cook J, et al. Strategies to improve recruitment to randomised controlled trials. *Cochrane Database Syst Rev* 2010;(4):MR000013. doi:10.1002/14651858.MR000013.pub5.

51. Hunt KJ, Shlomo N, Addington-Hall J. Participant recruitment in sensitive surveys: a comparative trial of 'opt in' versus 'opt out' approaches. *BMC Med Res Methodol* 2013;13:3. doi:10.1186/1471-2288-13-3.

52. Khalifa SAM, Swilam MM, El-Wahed AAA, et al. Beyond the pandemic: COVID-19 pandemic changed the face of life. *Int J Environ Res Public Health* 2021;18(11):5645. doi:10.3390/ijerph18115645.

53. Adams EL, Smith D, Caccavale LJ, et al. Parents are stressed! Patterns of parent stress across COVID-19. *Front Psychiatry* 2021;12:626456. doi:10.3389/fpsyt.2021.626456.

54. Darlington AE, Morgan JE, Wagland R, et al. COVID-19 and children with cancer: parents' experiences, anxieties and support needs. *Pediatr Blood Cancer* 2021;68(2):e28790. doi: 10.1002/pbc.28790.

55. Klosky JL, Tyc VL, Lawford J, et al. Predictors of non-participation in a randomized intervention trial to reduce environmental tobacco smoke (ETS) exposure in pediatric cancer patients. *Pediatr Blood Cancer* 2009;52(5):644-9. doi:10.1002/pbc.21946.

56. Farrand P, Woodford J, Coumoundouros C, et al. Supported cognitive-behavioural therapy self-help versus treatment-as-usual for depressed informal caregivers of stroke survivors (CEDArS): feasibility randomised controlled trial. *Cogn Behav Therap* 2020;13:e23. doi:10.1017/S1754470X20000239

For peer review only

57. Ammari AB, Hendriksen C, Rydahl-Hansen S. Recruitment and reasons for non-participation in a family-coping-orientated palliative home care trial (FamCope). *J Psychosoc Oncol* 2015;33(6):655-74. doi:10.1080/07347332.2015.1082168.
58. Bjertnaes O, Iversen HH, Skrivarhaug T. A randomized comparison of three data collection models for the measurement of parent experiences with diabetes outpatient care. *BMC Med Res Methodol* 2018;18(1):95. doi:10.1186/s12874-018-0557-z.
59. Shih T-H, Fan X. Comparing response rates from web and mail surveys: a meta-analysis. *Field Methods* 2008;20(3):249-271. doi:10.1177/1525822X08317085
60. Daikeler J, Bošnjak M, Lozar Manfreda K, Web versus other survey modes: an updated and extended meta-analysis comparing response rates. *J Surv Stat Methodol* 2020;8(3):513-539. doi:10.1093/jssam/smz008
61. Acquisti A, Brandimarte L, Loewenstein G. Secrets and likes: the drive for privacy and the difficulty of achieving it in the digital age. *J Consum Psychol* 2020;30(4):736-758. doi:10.1002/jcpy.1191
62. Robles T, Bordel B, Alcarria R, et al. Enabling trustworthy personal data protection in eHealth and well-being services through privacy-by-design. *Int J Distrib Sens Netw* 2020;16(5). doi:10.1177/1550147720912110.
63. Nanninga M, Reijneveld SA, Knorth EJ, et al. Expectations of barriers to psychosocial care: views of parents and adolescents in the community. *Eur Child Adolesc Psychiatry*. 2016;25(1):107-17. doi:10.1007/s00787-015-0717-1.
64. Zuidgeest M, Hendriks M, Koopman L, et al. A comparison of a postal survey and mixed-mode survey using a questionnaire on patients' experiences with breast care. *J Med Internet Res* 2011;13(3):e68. doi:10.2196/jmir.1241.
65. Alvarez-Galvez J, Salinas-Perez JA, Montagni I, et al. The persistence of digital divides in the use of health information: a comparative study in 28 European countries. *Int J Public Health* 2020;65(3):325-333. doi:10.1007/s00038-020-01363-w.
66. van Deursen AJ, van Dijk JA. Internet skills performance tests: are people ready for eHealth? *J Med Internet Res* 2011;13(2):e35. doi:10.2196/jmir.1581.
67. Mathiasen K, Riper H, Andersen TE, et al. Guided internet-based cognitive behavioral therapy for adult depression and anxiety in routine secondary care: observational study. *J Med Internet Res* 2018;20(11):e10927. doi:10.2196/10927.
68. Brown JSL, Murphy C, Kelly J, et al. How can we successfully recruit depressed people? Lessons learned in recruiting depressed participants to a multi-site trial of a brief depression intervention (the 'CLASSIC' trial). *Trials* 2019;20(1):131. doi:10.1186/s13063-018-3033-5.
69. Hughes-Morley A, Young B, Hempel RJ, et al. What can we learn from trial decliners about improving recruitment? Qualitative study. *Trials* 2016;17(1):494. doi:10.1186/s13063-016-1626-4.

For peer review only - http://bmjopen.bmj.com/site/about/guidelines.xhtml

70. van Warmerdam J, Zabih V, Kurdyak P, et al. Prevalence of anxiety, depression, and posttraumatic stress disorder in parents of children with cancer: a meta-analysis. *Pediatr Blood Cancer* 2019;66(6):e27677. doi:10.1002/pbc.27677.
71. Wikman A, Mattsson E, von Essen L, et al. Prevalence and predictors of symptoms of anxiety and depression, and comorbid symptoms of distress in parents of childhood cancer survivors and bereaved parents five years after end of treatment or a child's death. *Acta Oncol* 2018;57(7):950-957. doi:10.1080/0284186X.2018.1445286.
72. Badr H, Krebs P. A systematic review and meta-analysis of psychosocial interventions for couples coping with cancer. *Psychooncology* 2013;22(8):1688-704. doi:10.1002/pon.3200.
73. Heckel L, Gunn KM, Livingston PM. The challenges of recruiting cancer patient/caregiver dyads: informing randomized controlled trials. *BMC Med Res Methodol* 2018;18(1):146. doi:10.1186/s12874-018-0614-7.
74. Cuijpers P, Quero S, Noma H, et al. Psychotherapies for depression: a network meta-analysis covering efficacy, acceptability and long-term outcomes of all main treatment types. W*orld Psychiatry* 2021;20(2):283-293. doi:10.1002/wps.20860.
75. Robinson H, Appelbe D, Dodd S, et al. Methodological challenges in web-based trials: update and insights from the relatives' education and coping toolkit trial. *JMIR Ment Health* 2020;7(7):e15878. doi:10.2196/15878.
76. Brantnell A, Woodford J, Baraldi E, et al. Views of implementers and nonimplementers of internet-administered cognitive behavioral therapy for depression and anxiety: survey of primary care decision makers in Sweden. *J Med Internet Res* 2020;22(8):e18033. doi:10.2196/18033.
77. Gulliver A, Calear AL, Sunderland M, et al. Consumer-guided development of an engagement-facilitation intervention for increasing uptake and adherence for self-guided web-based mental health programs: focus groups and online evaluation survey. *JMIR Form Res* 2020;4(10):e22528. doi:10.2196/22528.
78. Nattinger AB, Pezzin LE, Sparapani RA, et al. Heightened attention to medical privacy: challenges for unbiased sample recruitment and a possible solution. *Am J Epidemiol* 2010;172(6):637-44. doi:10.1093/aje/kwq220.
79. Ward HJ, Cousens SN, Smith-Bathgate B, et al. Obstacles to conducting epidemiological research in the UK general population. *BMJ* 2004;329(7460):277-9. doi:10.1136/bmj.329.7460.277.
80. Halbesleben JR, Whitman MV. Evaluating survey quality in health services research: a decision framework for assessing nonresponse bias. *Health Serv Res* 2013;48(3):913-30. doi: 10.1111/1475-6773.12002.

For peer review only - http://bmjopen.bmj.com/site/about/guidelines.xhtml

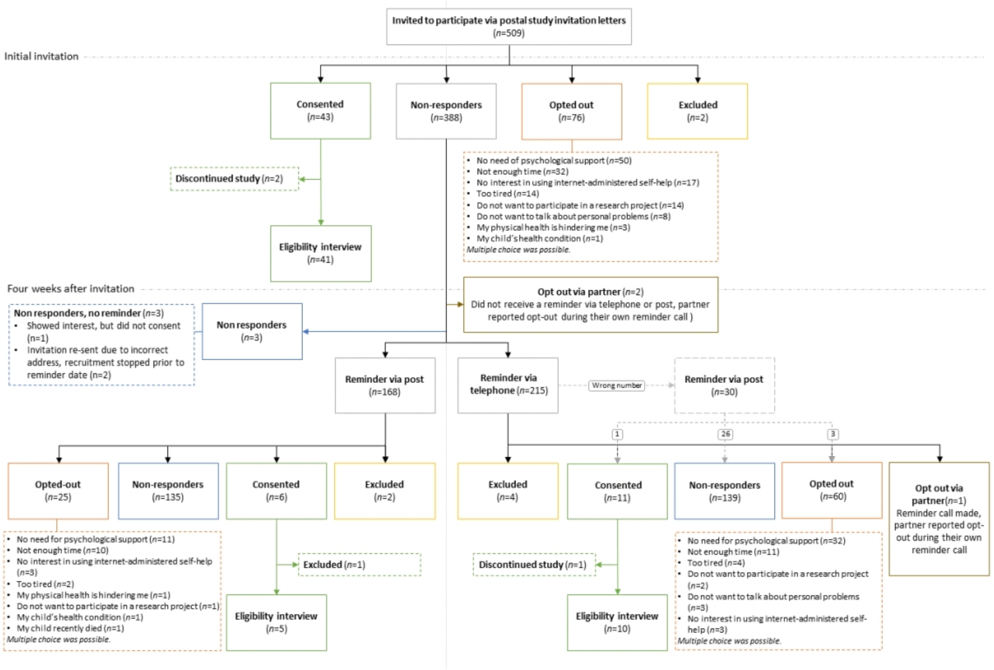

Figure 1. Recruitment flow via postal study invitation letters

909x631mm (38 x 38 DPI)

Supplement 3: Checklist for Reporting Of Survey Studies (CROSS)

Section/topic	Item	Item description	Reported on page #
**Title and abstract**			
Title and abstract	1a	State the word "survey" along with a commonly used term in title or abstract to introduce the study's design.	1, 2
	1b	Provide an informative summary in the abstract, covering background, objectives, methods, findings/results, interpretation/discussion, and conclusions.	2
**Introduction**			
Background	2	Provide a background about the rationale of study, what has been previously done, and why this survey is needed.	4-5
Purpose/aim	3	Identify specific purposes, aims, goals, or objectives of the study.	5
**Methods**			
Study design	4	Specify the study design in the methods section with a commonly used term (e.g., cross-sectional or longitudinal).	5
Data collection methods	5a	Describe the questionnaire (e.g., number of sections, number of questions, number and names of instruments used).	7-8
	5b	Describe all questionnaire instruments that were used in the survey to measure particular concepts. Report target population, reported validity and reliability information, scoring/classification procedure, and reference links (if any).	N/A
	5c	Provide information on pretesting of the questionnaire, if performed (in the article or in an online supplement). Report the method of pretesting, number of times questionnaire was pre-tested, number and demographics of participants used for pretesting, and the level of similarity of demographics between pre-testing participants and sample population.	N/A
	5d	Questionnaire if possible, should be fully provided (in the article, or as appendices or as an online supplement).	N/A
Sample characteristics	6a	Describe the study population (i.e., background, locations, eligibility criteria for participant inclusion in survey, exclusion criteria).	6
	6b	Describe the sampling techniques used (e.g., single stage or multistage sampling, simple random sampling, stratified sampling, cluster sampling, convenience sampling). Specify the locations of sample participants whenever clustered sampling was applied.	6
	6c	Provide information on sample size, along with details of sample size calculation.	7
	6d	Describe how representative the sample is of the study population (or target population if possible), particularly for population-based surveys.	18
Survey	7a	Provide information on modes of questionnaire administration, including the type and number of contacts, the location where the survey was conducted (e.g., outpatient	6

For peer review only

administration		room or by use of online tools, such as SurveyMonkey).	
	7b	Provide information of survey's time frame, such as periods of recruitment, exposure, and follow-up days.	9
	7c	Provide information on the entry process:<br>–>For non-web-based surveys, provide approaches to minimize human error in data entry.<br>–>For web-based surveys, provide approaches to prevent "multiple participation" of participants.	N/A
Study preparation	8	Describe any preparation process before conducting the survey (e.g., interviewers' training process, advertising the survey).	N/A
Ethical considerations	9a	Provide information on ethical approval for the survey if obtained, including informed consent, institutional review board [IRB] approval, Helsinki declaration, and good clinical practice [GCP] declaration (as appropriate).	18
	9b	Provide information about survey anonymity and confidentiality and describe what mechanisms were used to protect unauthorized access.	6
Statistical analysis	10a	Describe statistical methods and analytical approach. Report the statistical software that was used for data analysis.	8
	10b	Report any modification of variables used in the analysis, along with reference (if available).	N/A
	10c	Report details about how missing data was handled. Include rate of missing items, missing data mechanism (i.e., missing completely at random [MCAR], missing at random [MAR] or missing not at random [MNAR]) and methods used to deal with missing data (e.g., multiple imputation).	N/A
	10d	State how non-response error was addressed.	N/A
	10e	For longitudinal surveys, state how loss to follow-up was addressed.	N/A
	10f	Indicate whether any methods such as weighting of items or propensity scores have been used to adjust for non-representativeness of the sample.	N/A
	10g	Describe any sensitivity analysis conducted.	N/A
**Results**			
Respondent characteristics	11a	Report numbers of individuals at each stage of the study. Consider using a flow diagram, if possible.	9-10
	11b	Provide reasons for non-participation at each stage, if possible.	11-15
	11c	Report response rate, present the definition of response rate or the formula used to calculate response rate.	9

For peer review only

	11d	Provide information to define how unique visitors are determined. Report number of unique visitors along with relevant proportions (e.g., view proportion, participation proportion, completion proportion).	N/A
Descriptive results	12	Provide characteristics of study participants, as well as information on potential confounders and assessed outcomes.	10-11
Main findings	13a	Give unadjusted estimates and, if applicable, confounder-adjusted estimates along with 95% confidence intervals and p-values.	10-11
	13b	For multivariable analysis, provide information on the model building process, model fit statistics, and model assumptions (as appropriate).	N/A
	13c	Provide details about any sensitivity analysis performed. If there are considerable amount of missing data, report sensitivity analyses comparing the results of complete cases with that of the imputed dataset (if possible).	N/A
**Discussion**			
Limitations	14	Discuss the limitations of the study, considering sources of potential biases and imprecisions, such as non-representativeness of sample, study design, important uncontrolled confounders.	18
Interpretations	15	Give a cautious overall interpretation of results, based on potential biases and imprecisions and suggest areas for future research.	15-18
Generalizability	16	Discuss the external validity of the results.	18
**Other sections**			
Role of funding source	17	State whether any funding organization has had any roles in the survey's design, implementation, and analysis.	19-20
Conflict of interest	18	Declare any potential conflict of interest.	20
Acknowledgements	19	Provide names of organizations/persons that are acknowledged along with their contribution to the research.	19

For peer review only

BMJ Open

Opt-out rates and reasons for non-participation in a single-arm feasibility trial (ENGAGE) of a guided internet-administered CBT-based intervention for parents of children treated for cancer: a nested cross-sectional survey

Journal:	*BMJ Open*
Manuscript ID	bmjopen-2021-056758.R1
Article Type:	Original research
Date Submitted by the Author:	23-Feb-2022
Complete List of Authors:	Hagström, Josefin; Uppsala University, Healthcare Sciences and e-Health, Department of Women's and Children's Health<br>Woodford, Joanne; Uppsala University, Healthcare Sciences and e-Health, Department of Women´s and Children´s Health<br>von Essen, Agnes; Uppsala University, Healthcare Sciences and e-Health, Department of Women's and Children's Health<br>Lähteenmäki, Paivi ; TYKS Turku University Hospital Department of Paediatrics and Adolescent Medicine; Karolinska Institute, Pediatric Oncology and Pediatric Surgery, Department of Women's and Children's Health<br>von Essen, Louise; Uppsala University, Healthcare Sciences and e-Health, Department of Women's and Children's Health
**Primary Subject Heading**:	Mental health
Secondary Subject Heading:	Oncology, Paediatrics, Research methods
Keywords:	Depression & mood disorders < PSYCHIATRY, STATISTICS & RESEARCH METHODS, QUALITATIVE RESEARCH, Paediatric oncology < ONCOLOGY

SCHOLARONE™
Manuscripts

BMJ

I, the Submitting Author has the right to grant and does grant on behalf of all authors of the Work (as defined in the below author licence), an exclusive licence and/or a non-exclusive licence for contributions from authors who are: i) UK Crown employees; ii) where BMJ has agreed a CC-BY licence shall apply, and/or iii) in accordance with the terms applicable for US Federal Government officers or employees acting as part of their official duties; on a worldwide, perpetual, irrevocable, royalty-free basis to BMJ Publishing Group Ltd ("BMJ") its licensees and where the relevant Journal is co-owned by BMJ to the co-owners of the Journal, to publish the Work in this journal and any other BMJ products and to exploit all rights, as set out in our licence.

The Submitting Author accepts and understands that any supply made under these terms is made by BMJ to the Submitting Author unless you are acting as an employee on behalf of your employer or a postgraduate student of an affiliated institution which is paying any applicable article publishing charge ("APC") for Open Access articles. Where the Submitting Author wishes to make the Work available on an Open Access basis (and intends to pay the relevant APC), the terms of reuse of such Open Access shall be governed by a Creative Commons licence – details of these licences and which Creative Commons licence will apply to this Work are set out in our licence referred to above.

Other than as permitted in any relevant BMJ Author's Self Archiving Policies, I confirm this Work has not been accepted for publication elsewhere, is not being considered for publication elsewhere and does not duplicate material already published. I confirm all authors consent to publication of this Work and authorise the granting of this licence.

For peer review only

Opt-out rates and reasons for non-participation in a single-arm feasibility trial (ENGAGE) of a guided internet-administered CBT-based intervention for parents of children treated for cancer: a nested cross-sectional survey

Josefin HAGSTRÖM[1#] (josefin.hagstrom@kbh.uu.se); Joanne WOODFORD[1#] (joanne.woodford@kbh.uu.se); Agnes von ESSEN[1] (agnes-von.essen@kbh.uu.se); Päivi LÄHTEENMÄKI[2,3] (paivi.lahteenmaki@ki.se); Louise von ESSEN[1*] (louise-von.essen@kbh.uu.se).

[#]Joint authorship

Corresponding author

*Professor Louise von Essen, Healthcare Sciences and e-Health, Department of Women's and Children's Health, Uppsala University. Dag Hammarskjölds väg 14B, 751 05 Uppsala, Sweden. e-mail: louise.von.essen@kbh.uu.se Telephone: +46 070-425 07 14

Affiliation

[1]Healthcare Sciences and e-Health, Department of Women´s and Children´s Health, Uppsala University, Uppsala, Sweden

[2]Department of Pediatric and Adolescent Medicine, Turku University Hospital and Turku University, Turku, Finland

[3]Pediatric Oncology and Pediatric Surgery, Department of Women's and Children's Health, Karolinska Institutet, Stockholm, Sweden

Word Count: 5049

For peer review only

ABSTRACT

Objectives: Difficulties with recruitment into clinical trials are common. An opt-out recruitment strategy, whereby potential participants can decline further contact about a study (opt-out), and non-responders are contacted, may facilitate participation. Primary objectives examined opt-out and consent rates, mode and time point of opt-out, and sociodemographic characteristics of those who opted out versus chose to participate in a single-arm feasibility trial (ENGAGE) of a guided, internet-administered, CBT-based intervention for parents of children treated for cancer. Secondary objectives examined reasons for non-participation.

Design: A cross-sectional survey nested within the ENGAGE feasibility trial.

Setting: The intervention was delivered from Uppsala University, with parents located throughout Sweden.

Participants: Potential participants were recruited three months to five years following their child ending treatment for cancer and were identified via their personal identification number (via the Swedish Childhood Cancer Registry and Swedish Tax Agency) and invited via postal invitation packs and could opt-out via post, online, telephone, or e-mail. Those who did not opt-out or consent, within four weeks, received up to five telephone calls and/or one postal reminder.

Results: Of 509 invited 164 (32.2%) opted out, 78 (47.6%) via post, 53 (32.3%) via telephone, 24 (14.6%) online, and 6 (3.7%) via e-mail, 88 (53.7%) opted out after at least one telephone call and/or postal reminder. There was a trend for parents with lower educational levels to opt-out. No need of psychological support, lack of time, and no interest in internet-administered self-help were frequently reported reasons for non-participation.

Conclusions: Results emphasise the importance of using different opt-out modes and suggest future research should consider how to widen study participation for parents with lower education levels. Self-identifying a need for psychological support and the acceptability of internet-administered self-help are important factors for participation and should be considered in future research to increase recruitment.

Trial registration: ISRCTN57233429.

For peer review only

STRENGTHS AND LIMITATIONS OF THIS STUDY

- Parents invited are a nationally representative sample of parents of children treated for cancer.
- The use of different modes of opt-out (e.g., post, online, telephone, or e-mail) has rarely been investigated.
- The use of closed and open questions allows for a broad understanding of reasons for non-participation which can inform strategies to increase recruitment rates to future clinical trials.
- The study was not designed to compare recruitment rates across different strategies and conclusions concerning the effectiveness of opt-out versus opt-in strategies cannot be drawn.
- We did not collect sociodemographic variables of non-responders and therefore it was not possible to examine whether the sample of parents who opted out, or chose to participate, are representative of the overall population in relation to key sociodemographic variables.

Key words

Cancer, Internet-administered CBT, Low-intensity CBT, Non-participation, Opt-out, Parents, Recruitment.

For peer review only - http://bmjopen.bmj.com/site/about/guidelines.xhtml

INTRODUCTION

Background

Difficulties with recruitment into clinical trials are common [1], significantly contributing to research waste.[2] Insufficient recruitment rates have been cited as the leading cause of clinical trials being terminated [3] and reviews have found only 56% of publically funded clinical trials reach their target sample size.[4] Difficulties with recruitment into clinical trials may result in delays to the generation of robust evidence to inform decisions to adopt interventions into routine healthcare.[5] Whilst there is an increased focus on identifying effective clinical trial recruitment strategies [6] current evidence is limited [7] with little high quality evidence to inform decisions concerning the use of different recruitment strategies in clinical trials.[6]

The ENGAGE feasibility trial [8,9] is a single-arm feasibility trial of a guided, internet-administered low-intensity cognitive behavioural therapy (LI-CBT)-based intervention, designed to target common mental health difficulties (e.g., depression and generalised anxiety disorder) in parents of children treated for cancer.[10] The ENGAGE feasibility trial examines clinical (e.g., intervention drop-out, adherence, and acceptability) and procedural (e.g., recruitment and retention) uncertainties. Examining recruitment uncertainties is important given the recruitment of participants into mental health intervention trials is particularly challenging.[11] Barriers may include a reluctance to seek help,[12] stigma,[13,14] and low perceived need for support.[15] Recruitment into clinical trials of e-mental health (e-MH) interventions (e.g. internet-administered CBT), has been identified as particularly challenging, [16] with barriers including lack of trust, poor digital health literacy, low confidence in using technology, low acceptability, and preferences for face-to-face interventions.[17-20]

Given challenges with recruitment into e-MH interventions trials, there is a need to examine the effectiveness of different recruitment strategies to increase recruitment rates. One strategy associated with increased recruitment rates and less biased samples is the opt-out strategy.[21] Potential participants invited into a study are provided with a method of actively declining contact with the research team (opting out) if they do not wish to participate in or receive additional study information. Those who do not opt out are contacted by the research team, for example via the telephone, to provide more study information. Contacting non-responders may result in increased recruitment rates in comparison to opt-in strategies, whereby potential participants must take action to signal willingness to participate to the research team.[1,7,11,22] An opt-out strategy may be particularly effective for e-MH intervention trials.[16] Mental health difficulties such as depression include lack of motivation, concentration difficulties, lack of belief in deserving intervention, and negative expectations concerning recovery, which have been found to be barriers to help-seeking.[16,23,24] An opt-out strategy may facilitate participation e.g., potential participants experiencing low levels of motivation or problems with self-initiating behaviour [25] are contacted by the research team and

For peer review only - http://bmjopen.bmj.com/site/about/guidelines.xhtml

provided with an opportunity to receive more information. Indeed, opt-out strategies been found to result in the recruitment of populations who are less healthy and more functionally impaired than opt-in strategies.[21] Whilst successfully used with adult and paediatric populations,[26] to the best of our knowledge, an opt-out strategy has not been used with parents of children treated for cancer.

Furthermore, literature is scarce concerning reasons for non-participation reported by parents of children treated for cancer. Wider literature suggests common reasons include lack of perceived personal benefit, time, and energy and preconceptions about research.[27,28] Whilst qualitative research with parents of children treated for cancer has been undertaken to inform the development of recruitment strategies for clinical trials,[29] to the best of our knowledge the population's reasons for non-participation have not been investigated. Understanding reasons for non-participation may enable the identification of modifiable barriers to participation (e.g., treatment preferences, burden of trial procedures) [14] and inform the development of recruitment strategies to help overcome these barriers.[14]

Aims and objectives

Primary objectives examined: (1) opt-out and consent rates; (2) mode of opt-out; (3) time point of opt out; and (4) sociodemographic characteristics of those who opted out versus chose to participate in the ENGAGE feasibility trial.[8,9] Secondary objectives examined reasons for non-participation.

METHODS AND ANALYSIS

Study design

A cross-sectional survey with closed and open questions examining opt-out rates and reasons for non-participation nested within the ENGAGE feasibility trial [8,9] reported, where applicable, in line with the Checklist for Reporting of Survey Studies (CROSS).[30]

Participants

Eligible participants were: (1) parents of a child diagnosed with cancer during childhood (0-18 years) who had completed cancer treatment three months to five years previously; (2) living in Sweden; (3) able to read and write Swedish; (4) able access to e-mail, the internet, and a mobile telephone and Bank ID; and (5) self-reporting a need for psychological support related to the child's cancer disease and treatment and a wish to work with the intervention. Parents were excluded if they: (1) had a self-reported or psychologist-assessed (based on the Mini-International Neuropsychiatric Interview, M.I.N.I.[31]) severe and enduring mental health difficulty; and/or misuse of alcohol, street drugs, and/or prescription medication; (2) were acutely suicidal; and/or (4) attended psychological treatment.

Recruitment

For peer review only - http://bmjopen.bmj.com/site/about/guidelines.xhtml

Potential participants were recruited using two strategies: (1) postal study invitation packs; and (2) online advertisements via cancer organisations and interest groups. In this publication recruitment procedures and data associated with postal study invitation packs is reported.

Personal identification numbers of children who completed cancer treatment three months to five years previously were obtained from the Swedish Childhood Cancer Registry and linked to the names and addresses of their parent(s) via NAVET, a population registry held by the Swedish Tax Agency. Parents were invited in blocks of 100 until the target sample size (N=50) was reached. The first block invited was pre-selected with parents of children treated for cancer who ended treatment near to five years selected. The following four blocks were randomly selected. A de-identified list of all parents provided by NAVET was prepared by a research team member. To ensure allocation concealment a member of the U-CARE web-development team, external to the research team, used a computer generated simple randomization procedure to allocate parents into recruitment blocks. Postal study invitation packs were sent to parents' home addresses and included: (1) a study invitation letter, signed by the principle investigator (LvE) and a parent research partner (PRP); (2) a study information sheet and link to a secure website, the U-CARE-portal (Portal, an in-house platform which hosted the study and is designed to deliver internet-administered psychological interventions and support the collection of research data.[32,33]); (3) a paper reply slip to register interest in participation; and (4) an opt-out form and reasons for non-participation survey; and (5) a freepost envelope. The ENGAGE feasibility trial also included a Study Within A Trial (SWAT) investigating the effect of personalised versus non-personalised study invitation letters on recruitment and retention,[9] and results will be published in a separate publication.

Parents could access study information, in text and video format, and provide informed consent via the Portal. Parents who wished to opt out could do so by completing an opt-out form and reasons for non-participation survey via the Portal or by paper via the post (see Outcomes). Parents could also opt out by telephoning or e-mailing the research team.

Procedure

Opt-out and reminder telephone calls

Parents could sign an online form via the Portal using BankID, a secure electronic citizen authentication system used in Sweden, and were presented with a short reasons for non-participation survey (see Outcomes). Alternatively, parents could complete a paper-based survey, provide their name and ink signature, and return via the post. Parents who opted out via telephone or e-mail were asked to optionally provide a reason for non-participation.

For peer review only - http://bmjopen.bmj.com/site/about/guidelines.xhtml

Parents who did not respond (e.g., opt out or provide consent) within four weeks upon the first postal study invitation pack, received up-to five telephone reminder calls over four weeks. Telephone numbers were identified using internet search engines. A maximum of five reminder calls were made over four weeks, intended to confirm receipt of the postal study invitation and provide an opportunity to ask questions, or opt out from the study and further contact. Voicemails were left if the voicemail inbox had a personal recording clearly stating the parent's name. Communication between the parent and research team was documented in a recruitment case report form (CRF). If a telephone number could not be identified, a study invitation reminder letter and study invitation pack was resent. Personal details of parents who opted out were deleted from the research database.

Informed consent, screening, and baseline

Parents who chose to participate provided informed consent via the Portal and were contacted by the research team to schedule an eligibility interview with a licenced psychologist over the telephone. All parents were required to complete an online Portal assessment at baseline, or telephone if preferred. Full study procedures can be found in the ENGAGE feasibility trial protocol.[8]

Intervention

The intervention is an internet-administered, guided, LI-CBT based intervention (the EJDeR intervention; intErnetbaserad sJälvhjälp för förälDrar till barn som avslutat en behandling mot canceR).[10] Following Phase I of the Medical Research Council's (MRC) complex interventions framework [34] development of EJDeR was informed by a systematic review,[35] qualitative interview studies,[36,37] a single-arm trial,[38] participatory action research,[39] and a cross-sectional online survey.[40] A Parent Research Partner (PRP) group of two mothers and two fathers of a child treated for cancer informed further refinements.[10] EJDeR is delivered on the Portal, and designed to be worked with over 12 weeks and consists of four modules: (1) Introduction and Psychoeducation; (2) Behavioural Activation; (3) Worry Management; and (4) Relapse Prevention.[10] Parents are guided by e-therapists, including an initial assessment session via a secure inbuilt video conferencing system or telephone call, weekly written messages via the Portal, and a "booster" session mid-way through the intervention, via video conferencing or telephone call.

Outcomes

An online and paper-based survey, consisting of 13 items, in Swedish. The survey comprises two subsections: (1) sociodemographic characteristics (4 items) and (2) reasons for non-participation (9 items) questionnaire. An open question at the end of the questionnaire offered an opportunity for parents to provide any reasons for non-participation.

Sociodemographic characteristics

For peer review only - http://bmjopen.bmj.com/site/about/guidelines.xhtml

Background and sociodemographic characteristics was collected from parents who opted out via the online or paper-based survey: (1) gender (male/female/other); (2) age (years); (3a) relationship status (yes/no); (3b) cohabitation status (yes/no); and (4) highest level of education (free-text). Parents opting out via the telephone or e-mail were not asked to provide sociodemographic characteristics.

Reasons for non-participation

Parents were asked a closed, multiple choice question regarding reasons for non-participation, reasons were informed by previous research, [41,42] and parents could select all applicable reasons: (1) lack of time; (2) physical health; (3) not wanting to participate in research; (4) not liking to talk about personal problems; (5) too tired; (6) not interested in internet-administered self-help; (7) no need for psychological help; (8) child's health condition; and (9) child has recently passed away. The open question at the end of the reasons for non-participation questionnaire was: "If you do not want to participate in the project for reasons other than those mentioned above, please write these below".

Whereby the parent opted out via telephone or e-mail, a CRF was completed by the research team. The CRF included the reason(s) for non-participation listed in the survey and a free-text box in response to the open question.

Sample size

No formal sample size calculation was made [43] however recommendations for assessing feasibility outcomes were followed [44] with an aim to recruit 50 parents. It was estimated approximately 600 parents would be invited to meet the sample size.[9]

Progression criteria

A priori progression criteria [45] were set, these can be found in the published protocol for the ENGAGE feasibility trial.[8] A criteria that ≥9% of the total population invited into the study via the Swedish Childhood Cancer Registry chose to participate was set.

Data analysis

Data analysis was performed in Excel and SPSS for Windows, version 27.0 (IBM Corporation). Recruitment flow via postal study invitation packs is illustrated using an adapted CONSORT flow diagram.[46] Descriptive statistics were used to describe numbers and proportions who; opted out; opted out via each mode (postal/online/telephone/e-mail) and at each time-point (before/after telephone and/or postal reminder/s); and responded to a multiple choice question regarding reasons for non-participation. Study sample sociodemographic characteristics are described using descriptive statistics. Chi-square test was used to examine differences regarding categorical variables, and t-tests were calculated for continuous variables.

For peer review only - http://bmjopen.bmj.com/site/about/guidelines.xhtml

An inductive qualitative content analysis [47] managed using Microsoft Excel and Word was adopted to analyse responses to the open question in the reasons for non-participation survey. Free-text responses were read by two authors (JH and AvE) to gain an overall understanding of content. Next, condensed meaning units were identified (e.g., text sharing a common meaning) and assigned a code by JH and AvE separately. JH reviewed initial meaning units and codes to identify any differences in coding, with differences discussed by JH, AvE, and a third author (JW) if required. Codes were sorted into categories and subcategories by JH and AvE separately. As categories were identified, some content was recognised as not relating to reasons for non-participation, but included important content not to be discarded, for example, gratitude for the research. Categories were subsequently classified into two themes: (1) Reasons for non-participation and (2) Content not related to reasons for non-participation. JH, AvE, and JW met regularly to discuss categories and subcategories and ensure content was mutually exclusive. All responses were subsequently re-read by JH to check agreement with identified categories and subcategories. JH and AvE jointly prepared brief descriptions for categories and subcategories. Trustworthiness was established using peer examination of a supervising researcher (JW), two coders (JH and AvE) working independently, and record keeping.[48]

Other reasons for non-participation provided by parents via telephone or e-mail to the research team and recorded on the recruitment CRF were also categorised by JH and AvE separately, with all categorisations discussed with JW to obtain consensus.

Patient and Public involvement

The PRP group (two mothers and two fathers) provided feedback on the design and wording of the study invitation letter, included within the study invitation pack. PRPs were sent a study invitation letter draft via e-mail and asked to comment on wording, content, format, and design, and whether to include a PRP's name and signature on the letter alongside that of the principle investigator (LvE). Parents did not suggest any changes and considered the letter to be concise, validating, and informative and agreed the name and signature of a PRP should be included.[9] Patient and public involvement may have been improved by engaging PRPs in drafting the opt-out survey, for example informing the options available in the multiple choice question regarding reasons for non-participation.

RESULTS

Opt-out and consent rates

Recruitment via postal study invitation packs into the ENGAGE feasibility trial took place between 3rd July 2020 and 30th November 2020. 509 parents were identified and invited. 32.2% (164/509) opted out, and 11.8% (60/509) provided consent, exceeding the a priori recruitment rate progression criteria

For peer review only - http://bmjopen.bmj.com/site/about/guidelines.xhtml

of ≥9% invited via the Swedish Childhood Cancer Registry.[8,45]. 11.0% (56/509) were found to be eligible for the study.

Three parents were initially registered as opting out via the telephone by a partner who had also received a study invitation pack. Subsequently, the opt-out protocol was adapted and opting out via a partner was no longer an option. Recruitment flow via postal study invitation packs can be seen in Figure 1.

-INSERT FIGURE 1 ABOUT HERE-

Mode of opting-out

The most frequent mode of opt-out was via the post (78/164, 47.6 %), followed by telephone (53/164, 32.3%), the Portal (24/164, 14.6%), and e-mail (6/164, 3.7%). Three parents (3/164; 1.8%) opted out via their partner over the telephone. Two who initially opted out via telephone, subsequently opted out via the post, resulting in a total of n=80 opt-outs via the post.

Time point of opting-out

The majority of opt-outs occurred after one or more telephone reminder call and/or postal reminder (88/164, 53.7%). The mean number of days between a postal study invitation pack being sent and an opt-out was 30.0 days (SD=20.6, range = 2-158).

Sociodemographic characteristics of parents who opted out versus choose to participate

Sociodemographic characteristics of parents (n=105) who opted out and provided sociodemographic data and parents (n=56) who consented and were screened for eligibility is shown in Table 1. No differences between groups were found, though there was a trend for parents with lower educational levels to opt out, and parents with a tertiary education to choose to participate.

For peer review only

Table 1. Sociodemographic characteristics for parents who opted out and who consented and were screened for eligibility

Characteristic	Opted out (n=105)[a]	Consented and screened for eligibility (n=56)	t value, $\chi 2$ value	p Value
**Age, mean (SD, range)**	44.8 (7.6, 29-68)	43.5 (7.2, 32-62)	-0.988	0.325
Missing (n, %)	19 (18.1)	0 (0)		
**Gender (n, %)**				
Female	54 (51.4)	33 (58.9)	0.001	0.978
Male	38 (36.2)	23 (41.1)		
Missing	13 (12.4)	0 (0)		
**Relationship status (n, %)**				
Partner	86 (81.9)	47 (83.9)	1.996	0.158
Single	8 (7.6)	9 (16.1)		
Missing	11 (10.5)	0 (0)		
If partner, cohabiting				
Yes	78 (90.7)	46 (97.9)	1.035	0.309
No	5 (5.8)	1 (2.1)		
Missing	3 (3.5)	0 (0)		
**Level of education (n, %)[b]**				
Lower secondary	6 (5.7)	1 (1.8)	6.085	0.298
Upper secondary	30 (28.6)	11 (19.6)		
Post-secondary non-tertiary	5 (4.8)	3 (5.4)		
Tertiary education	50 (47.6)	40 (71.4)		
PhD	2 (1.9)	1 (1.8)		
Not possible to categorize	1 (1.0)	0 (0)		
Missing	11 (10.5)	0 (0)		

[a]105 parents who opted out provided some sociodemographic data. Sociodemographic characteristics were collected from parents who opted out by paper via the post (n=80) or Portal (n=24). One parent opted out via e-mail and voluntarily provided some sociodemographic data.

[b]Level of education was self-reported as a free-text response, with responses categorised by research team.

Reasons for non-participation

Closed question

83.5% (137/164) responded to the closed question about reasons for non-participation. The most frequently reported reason was not experiencing any need for psychological support (93/137, 67.9%)

For peer review only

followed by not having enough time (53/137, 38.7%), and not interested in using internet-administered self-help (23/137, 16.8%). Responses are shown in Table 2 and presented separately for female and male gender where data was available.

Table 2. Reasons for non-participation in response to the closed multiple-choice question

Reasons	Female (n=50[a])		Male (n=35[a])		Total (n=137[b])	
	n	%	n	%	n	%
I do not experience any need for psychological support	32	64.0	27	77.1	93	67.9
I do not have enough time	24	48.0	19	54.3	53	38.7
I am not interested in using internet-administered self-help	10	20.0	8	22.9	23	16.8
I am too tired to participate in a research project	9	18.0	6	17.1	20	14.6
I do not want to participate in a research project	7	14.0	8	22.9	17	12.4
I do not like talking about my personal problems	5	10.0	4	11.4	11	8.0
My physical health is hindering me	3	6.0	1	2.9	4	2.9
I do not want to participate because of my child's health condition	2	4.0	0	0	2	1.5
I do not want to participate because my child recently died	1	2.0	0	0	1	0.7

[a]n=85 parents provided data regarding gender (female/male)

Open question

Thirty parents responded to the open question regarding reasons for non-participation in the paper-based or online survey. 21 provided a response via telephone or e-mail recorded in the CRF. Content analysis of responses within the theme Reasons for non-participation resulted in seven categories: Timing, Emotional wellbeing, Support, Perceived ineligibility, Other commitments, and Reluctance to engage in internet-administered self-help. Responses within the theme Content not related to reasons for non-participation were categorised as: Not for me, but…. and When my child was ill. Themes, categories, subcategories, and quotations are shown in Table 3.

For peer review only - http://bmjopen.bmj.com/site/about/guidelines.xhtml

Table 3. Themes, categories, subcategories, and quotations. Categories are presented in order of number of included responses whereas subcategories are presented alphabetically

Theme	Category	N (n=51)	Subcategory	Quotation (P: reported by parent; R: recorded by researcher)
Reasons for non-participation	Timing	20	Earlier need	“We would have needed help a long time ago, but we have managed well and right now we are doing well” (P)
				“The need was bigger right after completed treatment” (R)
			Not right now	”Could be interesting another time” (P)
	Emotional wellbeing	16	Coping strategies	“We/I don’t have the energy to be involved in sickness anymore, more than I am” (P)
				“Sure, it was heavy and tough, but life is what you make it” (P)
			Family wellbeing	”… my son is well today, and us parents as well” (P)
				”Child is doing fine” (R)
			Fatigue	“We are tired and it’s always a lot, but it’s not related to the cancer [the child’s]” (P)
			No need	“Feeling no need for help, feeling good” (P)
				“Felt no need for therapy” (R)
	Support	13	Professional support	“I have processed a lot on my ‘own’ with the help from a psychologist” (P)
				“Has received psychological treatment” (R)
			Social support	”[I’ve come a long way] with help from […] and close ones” (P)
				“Had a good social network helping the family” (R)
	Perceived ineligibility	11	N/A	“Cannot read Swedish” (P)
				“In psychological treatment” (R)
				“I’m too broken in many ways, and unfortunately I cannot participate” (P)
				“Did not feel suitable for the project” (R)

For peer review only

For peer review only - http://bmjopen.bmj.com/site/about/guidelines.xhtml

	Other commitments	7	N/A	"Unfortunately I don't have the time to participate" (P)<br>"Too busy to participate" (R)
	Reluctance to engage in internet-administered self-help	2	N/A	"Did not feel comfortable with the internet" (R)
Content not related to reasons for non-participation	Not for me, but …	19	Gratitude	"Great that you are doing this" (P)<br>"Very positive about project" (R)
			Need among others	"I hope many participate, there is really a need for support after treatment" (P)<br>"Would love to help other parents" (R)
	When my child was ill	10	Available care	"The care after completing treatment was non-existent" (R)
			Cancer illness	"My son was critically ill when the intestine broke" (P)
			Family life	"When [child] was diagnosed with cancer, my husband and I tried to keep things as 'normal' as possible" (P)

Reasons for non-participation

The category Timing includes the subcategories: *Earlier need* and *Not right now*. *Earlier need* concerns the invitation coming "too late" i.e. after treatment completion, with parents positing hypothetical participation had they been approached earlier. *Not right now* concerns not currently interested in participation, however potential interest another time.

The category Emotional wellbeing includes the subcategories: *Coping strategies*, *Family wellbeing, Fatigue,* and *No need*. *Coping strategies* concerns use of coping strategies. Parents stated wanting to forget "what has been" (e.g., the cancer experience) and a reluctance to "go back and think about these things" or to remain involved with sickness. Conversely, parents reported focusing on the positive, accepting their situation, holding a "positive" attitude towards difficult situations, and post-traumatic growth. Parents could report distancing from the cancer experience in the past and present as well as a want to "focus on the positive" in the future. *Family wellbeing* concerns the entire family, for example as their child was in good health, psychological support for the parent was not considered needed. *Fatigue* concerns being too tired to participate and *No need* concerns parents feeling well and not experiencing a need for psychological support.

For peer review only

The category Prior support includes the subcategories: *Professional support* and *Social support*. *Professional support* concerns receipt of current or past support from a health professional, for example, a psychologist or counsellor whereas *Social support* concerns support and help by family, friends, and pets.

The category Perceived ineligibility concerns parents not considering themselves eligible for the study, e.g. not fulfilling the inclusion criteria for example insufficient knowledge of Swedish, undergoing psychological treatment as well as other reasons such as being “too broken” to participate.

The category Other Commitments concerns life commitments occupying parents’ time, reference was also made to too many invitations regarding participation in research and a wish to have free time.

The category Reluctance to engage in an internet-administered intervention included responses whereby parents reported being uncomfortable with the internet or preferring face-to-face treatment.

Content not related to reasons for non-participation

The category Not for me, but… includes the subcategories: *Gratitude* and *Need among others*. *Gratitude* concerns the importance of the study and gratitude that it was carried out. *Need among others* concerns a hope that other parents would participate and receive help, and that there is a need for support for parents after their child’s cancer treatment.

The category When my child was ill includes the subcategories: *Available care*, *Cancer illness,* and *Family life*. *Available care* concerns the care during the child’s illness and treatment. Parents mentioned there had been little care available for their wellbeing after their child had completed treatment. *Cancer illness* concerns details about the child’s illness and treatment, for example the duration of the treatment or the child having been ill since birth. *Family life* concerns experiences during their child’s illness and treatment, for example, striving for normality or difficult family events such as separating from a partner.

DISCUSSION

Summarising the main findings, the opt-out rate was 32.2%, the consent rate via postal study invitation packs was 11.8%, and 11.0% were eligible for the trial. The most frequent mode of opt-out was by paper via the post (47.6 %). The majority (53.7%) opted out of further contact with the research team after one or more reminder via telephone and/or post. Although not significant, there was a trend for parents with lower educational levels to opt out, and parents with a tertiary education choosing to participate. Common reasons for participation in response to the closed question were not experiencing any need for psychological support not having enough time, and not having an interest in

For peer review only - http://bmjopen.bmj.com/site/about/guidelines.xhtml

using internet-administered self-help. Content analysis of responses to the open question agreed with results from the closed question, with reasons for non-participation including emotional wellbeing, receipt of psychological support, and commitments limiting time to participate. Additional reasons were suggested, including study invitation timing, and parents not perceiving themselves as eligible.

The overall consent rate exceeded the recruitment target outlined in study progression criteria.[8,45] Use of an opt-out strategy has demonstrated higher recruitment rates when compared to opt-in strategies.[7,49,50] For example, randomised controlled trials (RCTs) comparing opt-out versus opt-in strategies have found recruitment rates of 40-50% for opt-out, versus 26-38% for opt-in.[21,51]. However, studies utilising opt-out strategies are highly heterogeneous, with variations in recruitment rates, populations, designs, and interventions [49] and few RCTs have compared opt-out versus opt-in strategies [7,50], limiting conclusions regarding effectiveness. The ENGAGE feasibility trial did not include an opt-in comparison arm, making it impossible to determine whether successful recruitment was a result of an opt-out strategy.

Recruitment took place during the COVID-19 pandemic. Given physical distancing recommendations in Sweden, as in many countries globally,[52] people have spent increased time at home. Research has observed increased parental stress associated with physical distancing measures and the closure of schools and childcare facilities.[53] A retrospective online survey conducted in the United States found that 71.1% of parents reported increases in parenting-specific stress from before COVID-19.[53] Further, a survey conducted in the United Kingdom indicated 85% of parents of children with cancer were worried about the virus, for example risk of infection, reduced healthcare provision, and increased risk of social isolation, and 70% no longer deemed the hospital a safe place.[54] The context of a pandemic may have facilitated recruitment, e.g., parents may have been experiencing elevated levels of fear and anxiety. Conversely, the pandemic may have been a barrier to participation for some, given parents of children with cancer commonly focus on their child's needs, at the expense of their own wellbeing.[35-37]

The overall opt-out rate in the present study is higher than a 19% opt-out rate found in a longitudinal cohort pilot study of angina patients [21] and a 21% opt-out rate seen in a survey study regarding end of life care.[51] However, existing literature on opt-out strategies tends to be on observational studies, as opposed to intervention or health service-orientated research [49] making it difficult to compare results with the wider literature. When comparing the opt-out rate in the present study to non-participation rates in intervention studies for parents or informal caregivers using opt-in strategies, findings are also varied. In an RCT of an intervention for parents of children with cancer to reduce exposure to tobacco smoke, 24% approached about study participation within a clinic setting declined participation [55] and in a pilot RCT of guided CBT self-help for informal caregivers of stroke

For peer review only - http://bmjopen.bmj.com/site/about/guidelines.xhtml

survivors, 15% invited by post actively declined participation.[56] Further, in an RCT of a psychological intervention for cancer patients in palliative care and their family caregivers 65.9% of potential participants actively declined participation.[57]

To the best of our knowledge, this is the first study to specifically examine the rate of opt-out across four different modes of opt-out. Commonly, studies utilising opt-out recruitment strategies include only one mode of opt-out, for example via the post [26; 58] or telephone.[59] The most frequent mode of opt-out in the ENGAGE feasibility trial was paper via the post (47.6 %), followed by telephone (32.3%), with findings similar to a quantitative telephone survey with military veterans examining rural and urban differences in attitudes towards mental health care and influence on mental health service use, whereby opt-out via the post was more frequent (75%) than opt-out via the telephone (25%).[49] Interestingly, only 14.6% of parents opted out online via the Portal in the ENGAGE feasibility trial. In comparison with the general adult population, parents of a child treated for cancer three months to five years following end of treatment may be assumed to be younger, with good digital knowledge and skills.[60] Therefore it may have been anticipated that parents would prefer opt-out online. However, systematic reviews and meta-analyses have shown postal survey response to be, in general, higher than online surveys.[61,62] One potential reason is rising concerns about security and privacy in the digital age.[63,64] Indeed, common barriers to engaging in internet-administered interventions include security and confidentiality concerns [19] Possibly, parents may have experienced opt-out via the post as implying higher levels of security and privacy than using an electronic personal identification method (BankID) to opt-out online. However, the utilisation of different modes of opt-out was easy to implement and given the variation in response rate across the four opt-out modes, future studies may wish to offer potential participants multiple modes of opt-out. Further, future studies could examine additional potential modes of opt-out, for example, via short message service (SMS).[65]

The trend for parents with an upper secondary school education to opt out of the study while parents with a tertiary education chose to participate, is supported by the wider literature. For example, parents of adolescents with psychosocial difficulties who have lower levels of education perceive more barriers towards accessing psychosocial care, than parents with a university level education.[66] Moreover, evidence suggests online survey responders tend to be more highly educated than non-responders [67] and adults with lower levels of education struggle to find health information online [68,69] and require more guidance to use internet-administered interventions.[69] Higher education levels are also associated with increased effectiveness and higher completion rates of guided internet-administered CBT interventions in a general adult population.[70] Future research is needed to improve the acceptability and feasibility of internet-administered interventions for people with lower education levels.

For peer review only - http://bmjopen.bmj.com/site/about/guidelines.xhtml

In line with previous research with depressed adult populations,[71,72] the most common reason for non-participation was not perceiving a need for psychological support. Similar findings were found in psychological intervention studies for adult cancer patient/caregiver dyads [3] and informal caregivers of stroke survivors.[56] Around 14-30% of parents of children treated for cancer report clinically relevant levels of mental health difficulties, such as depression, anxiety, and posttraumatic stress.[31,73,74] Therefore at a group level, a majority of parents report levels of psychological distress within a normal range [35] and it can be anticipated most do not experience a need for psychological support. However, as the 11.8% recruitment rate is under the prevalence rate of psychological distress in the population, some parents in need of psychological support did not consent and research to increase participation among this group is warranted.

Study invitation timing was the most commonly cited reason for non-participation in response to the open question, corroborating findings of a similar study conducted with parents of children with cancer examining potential barriers to recruitment.[29] Parents found it difficult to identify the most appropriate time point for intervention, as periods of increased distress may differ between parents.[29] Other research has found invitation timing plays a significant role in a person's decision to participate in studies of psychosocial interventions for couples coping with cancer [75] and that it is difficult to find an optimal time point to recruit cancer patient/caregiver dyads into intervention research.[76] A potential solution may be to not place any time limit from diagnosis/ end of treatment.

Finally, lack of interest in internet-administered self-help was a common reason for non-participation. Despite well demonstrated clinical effectiveness of e-MH interventions[77], difficulties with recruitment into trials are common [78] and rates of implementation are low.[79] Barriers include lack of trust, poor digital health literacy, low levels of confidence in using digital technology, and preferences for face-to-face interventions.[17-20] Research indicates acceptability may increase when potential users are provided with information about intervention content and/or effectiveness [18,80] and future research may wish to examine the provision of additional intervention information, including for example screenshots or videos, on recruitment rates.

Ethical considerations

The study was approved by the Regional Ethical Review Board in Uppsala, Sweden (Dnr: 2017/527) and was conducted in accordance with the Helsinki Declaration and Good Clinical Practice (GCP) guidelines.

One concern around an opt-out strategy is the use of repeated reminders/contact attempts, and potentially coercing participants.[21] While research ethics committees often require researchers to utilise opt-in recruitment strategies to protect participant confidentiality and (medical) privacy [81],

For peer review only - http://bmjopen.bmj.com/site/about/guidelines.xhtml

this minimises the opportunity for researchers to speak with potential participants directly to explain the study.[82] Consistent with other research utilising opt-out recruitment whereby few concerns have been raised,[81] only one complaint concerning reminder calls was received during the study, indicating an opt-out recruitment strategy is acceptable to the population.

Strengths and limitations

This study has several strengths. First, parents were identified and randomly invited into the study via the Swedish Childhood Cancer Registry and a population registry held by the Swedish Tax Agency (NAVET). As such, invited parents are a nationally representative sample of parents of children treated for cancer in Sweden. Second, parents were provided with different modes of opt-out (e.g., post, online, telephone, or e-mail). To the best of our knowledge, this is the first study to compare opt-out rates across different opt-out modalities. Third, by using both closed and open questions to examine reasons for non-participation, results provide us with a broad understanding of reasons for non-participation which may inform the development of future recruitment strategies. The study also has limitations. First, it was not designed to compare recruitment rates across different recruitment strategies and conclusions on the effectiveness of the opt-out strategy versus other recruitment strategies cannot be drawn. Future studies may look to investigate the effect of an opt-out recruitment strategy with an opt-in recruitment strategy on recruitment rates. Second, we did not collect sociodemographic variables of non-responders and therefore it was not possible to assess whether the sample is representative of the overall population in relation to key sociodemographic variables. Some sociodemographic data (e.g., sex, age, geographical location) could have been obtained for all potential participants via the NAVET registry, which would have enabled comparisons between participants who consented, opted out, and were non-responders [83] however we did not include the collection of this data in the ethics application. Third, whilst the open question regarding reasons for non-participation allowed for a broad understanding of reasons for non-participation, embedding semi-structured interviews to explore reasons for non-participation in more depth, may have facilitated a richer understanding of reasons for non-participation in the population.

CONCLUSIONS

This is the first study to examine the use of an opt-out strategy and different modes of opt-out within a feasibility trial examining the feasibility and acceptability of an internet-administered, guided, LICBT based self-help intervention for parents of children treated for cancer. Though recruitment into clinical trials is challenging, projected recruitment rates were exceeded. Despite the most frequent mode of opt-out being paper via the post, given the implementation of different modes of opt-out was straightforward, coupled with the variation in response rate across the four opt-out modes, future studies should consider using multiple modes of opt-out. Developing a more comprehensive understanding of reasons for non-participation, may help inform planning and decision-making

For peer review only - http://bmjopen.bmj.com/site/about/guidelines.xhtml

concerning study design, and potentially enhance recruitment rates and reduce research waste in future trials of internet-administered interventions for both parent/caregiver and wider populations. Future research may wish to compare opt-out versus opt-in strategies within an RCT and embed qualitative interviews to explore reasons for non-participation and develop a more in-depth understanding of potential barriers to trial participation.

ACKNOWLEDGEMENTS

The authors thank the four PRPs (Helena Börjesson, Martin Hedqvist, Anna Mautner, and Erik Olsson) for their feedback on study invitation material. In addition, we wish to thank Fabian Holmberg, Ian Horne, and Ylva Hägg Sylvén (Portal team members) for their assistance with developing the opt-out procedure on the Portal. We also thank research assistant, Christina Reuter, for assisting with some elements of data preparation and analysis.

AUTHORS' CONTRIBUTIONS

Author contributions are written in accordance with the CRediT statement: JH: formal analysis, investigation, data curation, writing - original draft, visualization; JW: methodology, formal analysis, writing – original draft, supervision, project administration; AvE: validation, investigation, data curation, formal analysis, writing – review and editing; PL: resources, writing – review and editing; LvE: conceptualization, methodology, resources, writing – review and editing, supervision, project administration, funding acquisition.

FUNDING

This work is supported by the Swedish Research Council (grant number K2015-99X-20836-08-4 and 2018-02578), the Swedish Cancer Society (grant number 150673 and 180589), the Swedish Childhood Cancer Foundation (grant number PR2017-0005), and funding via the Swedish Research Council to U-CARE, a Strategic Research environment (Dnr 2009–1093). The funders had no role in study design, data collection and analysis, decision to publish, or preparation of the manuscript.

COMPETING INTERESTS

The authors have no competing interests to declare

ABBREVIATIONS

CBT: Cognitive Behavioural Therapy

CRF: Case Report Form

For peer review only - http://bmjopen.bmj.com/site/about/guidelines.xhtml

e-MH: e-Mental Health

LI-CBT: Low-Intensity Cognitive Behavioural Therapy

MRC: Medical Research Council

FIGURES

Figure 1. Recruitment flow via postal study invitation packs

DATA AVAILABILITY STATEMENT

The research data supporting the findings of this study is stored in Zenodo repository with the identifier https://doi.org/10.5281/zenodo.6136920. Access to the data stored in Zenodo is available upon written request from the corresponding author (LvE).

REFERENCES

1. Bower P, Brueton V, Gamble C, et al. Interventions to improve recruitment and retention in clinical trials: a survey and workshop to assess current practice and future priorities. *Trials* 2014;15:399. doi:10.1186/1745-6215-15-399.
2. Moher D, Glasziou P, Chalmers I, et al. Increasing value and reducing waste in biomedical research: who's listening? *Lancet* 2016;387(10027):1573-1586. doi:10.1016/S0140-6736(15)00307-4.
3. Williams RJ, Tse T, DiPiazza K, et al. Terminated trials in the ClinicalTrials.gov results database: evaluation of availability of primary outcome data and reasons for termination. *PLoS One* 2015;10(5):e0127242. doi:10.1371/journal.pone.0127242.
4. Walters SJ, Bonacho Dos Anjos Henriques-Cadby I, Bortolami O, et al. Recruitment and retention of participants in randomised controlled trials: a review of trials funded and published by the United Kingdom Health Technology Assessment Programme. *BMJ Open* 2017;7(3):e015276. doi:10.1136/bmjopen-2016-015276.
5. Richards DA, Ross S, Robens S, et al. The DiReCT study - improving recruitment into clinical trials: a mixed methods study investigating the ethical acceptability, feasibility and recruitment yield of the cohort multiple randomised controlled trials design. *Trials* 2014;15:398. doi:10.1186/1745-6215-15-398.
6. Treweek S, Bevan S, Bower P, et al. Trial Forge Guidance 1: what is a Study Within A Trial (SWAT)? *Trials* 2018;19(1):139. doi:10.1186/s13063-018-2535-5.
7. Treweek S, Pitkethly M, Cook J, et al. Strategies to improve recruitment to randomised trials. *Cochrane Database Syst Rev* 2018;2(2):MR000013. doi:10.1002/14651858.MR000013.pub6.
8. Woodford J, Wikman A, Cernvall M, et al. Study protocol for a feasibility study of an internet-administered, guided, CBT-based, self-help intervention (ENGAGE) for parents of children

For peer review only

previously treated for cancer. *BMJ Open* 2018;8(6):e023708. doi:10.1136/bmjopen-2018-023708.

9. Woodford J, Norbäck K, Hagström J, et al. Study within a trial (SWAT) protocol. Investigating the effect of personalised versus non-personalised study invitations on recruitment: An embedded randomised controlled recruitment trial. *Contemp Clin Trials Commun* 2020;18:100572. doi:10.1016/j.conctc.2020.100572.
10. Woodford J, Farrand P, Hagström J, et al. Internet-administered cognitive behavioral therapy for common mental health difficulties in parents of children treated for cancer: intervention development and description study. *JMIR Form Res* 2021;5(7):e22709. doi:10.2196/22709.
11. Liu Y, Pencheon E, Hunter RM, et al. Recruitment and retention strategies in mental health trials - a systematic review. *PLoS One* 2018;13(8):e0203127. doi:10.1371/journal.pone.0203127.
12. Mackenzie CS, Erickson J, Deane FP, et al. Changes in attitudes toward seeking mental health services: a 40-year cross-temporal meta-analysis. *Clin Psychol Rev* 2014;34(2):99-106. doi:10.1016/j.cpr.2013.12.001.
13. Clement S, Schauman O, Graham T, et al. What is the impact of mental health-related stigma on help-seeking? A systematic review of quantitative and qualitative studies. *Psychol Med* 2015;45(1):11-27. doi:10.1017/S0033291714000129.
14. Hughes-Morley A, Young B, Waheed W, et al. Factors affecting recruitment into depression trials: Systematic review, meta-synthesis and conceptual framework. *J Affect Disord* 2015;172:274-90. doi:10.1016/j.jad.2014.10.005.
15. Mojtabai R, Olfson M, Sampson NA, et al. Barriers to mental health treatment: results from the National Comorbidity Survey Replication. *Psychol Med* 2011;41(8):1751-61. doi:10.1017/S0033291710002291.
16. Woodford J, Farrand P, Bessant M, et al. Recruitment into a guided internet based CBT (iCBT) intervention for depression: lesson learnt from the failure of a prevalence recruitment strategy. *Contemp Clin Trials* 2011;32(5):641-8. doi:10.1016/j.cct.2011.04.013.
17. Apolinário-Hagen J, Kemper J, Stürmer C. Public acceptability of e-mental health treatment services for psychological problems: a scoping review. *JMIR Ment Health* 2017;4(2):e10. doi: 10.2196/mental.6186.
18. March S, Day J, Ritchie G, et al. Attitudes toward e-mental health services in a community sample of adults: online survey. *J Med Internet Res* 2018;20(2):e59. doi:10.2196/jmir.9109.
19. Schreiweis B, Pobiruchin M, Strotbaum V, et al. Barriers and facilitators to the implementation of eHealth services: systematic literature analysis. *J Med Internet Res* 2019;21(11):e14197. doi:10.2196/14197.

For peer review only

For peer review only - http://bmjopen.bmj.com/site/about/guidelines.xhtml

20. Vis C, Mol M, Kleiboer A, et al. Improving implementation of eMental Health for mood disorders in routine practice: systematic review of barriers and facilitating factors. *JMIR Ment Health* 2018;5(1):e20. doi:10.2196/mental.9769.

21. Junghans C, Feder G, Hemingway H, et al. Recruiting patients to medical research: double blind randomised trial of "opt-in" versus "opt-out" strategies. *BMJ* 2005;331(7522):940. doi:10.1136/bmj.38583.625613.AE.

22. Trevena L, Irwig L, Barratt A. Impact of privacy legislation on the number and characteristics of people who are recruited for research: a randomised controlled trial. *J Med Ethics* 2006;32(8):473-7. doi:10.1136/jme.2004.011320.

23. Roberts T, Miguel Esponda G, Krupchanka D, et al. Factors associated with health service utilisation for common mental disorders: a systematic review. *BMC Psychiatry* 2018;18(1):262. doi:10.1186/s12888-018-1837-1.

24. Shi W, Shen Z, Wang S, et al. Barriers to professional mental health help-seeking among Chinese adults: a systematic review. *Front Psychiatry* 2020;11:442. doi:10.3389/fpsyt.2020.00442.

25. Katon WJ. Epidemiology and treatment of depression in patients with chronic medical illness. Dialogues *Clin Neurosci* 2011;13(1):7-23. doi:10.31887/DCNS.2011.13.1/wkaton.

26. McCullough MB, Janicke D, Odar Stough C, et al. Barriers to recruitment in pediatric obesity trials: comparing opt-in and opt-out recruitment approaches. *J Pediatr Psychol* 2017;42(2):174-185. doi:10.1093/jpepsy/jsw054.

27. Nalubega S, Evans C. Participant views and experiences of participating in HIV research in sub-Saharan Africa: a qualitative systematic review. *JBI Database System Rev Implement Rep* 2015;13(5):330-420. doi:10.11124/jbisrir-2015-2051.

28. Rivers D, August EM, Sehovic I, et al. A systematic review of the factors influencing African Americans' participation in cancer clinical trials. *Contemp Clin Trials* 2013;35(2):13-32. doi:10.1016/j.cct.2013.03.007.

29. Canter KS, Vega G, Ramirez AP, et al. Strategies for successful recruitment and retention of parents in pediatric psychosocial eHealth interventions: a qualitative study in pediatric oncology. *J Pediatr Psychol* 2020;45(5):530-539. doi:10.1093/jpepsy/jsaa013.

30. Sharma A, Minh Duc NT, Luu Lam Thang T, et al. A Consensus-Based Checklist for Reporting of Survey Studies (CROSS). *J Gen Intern Med* 2021. doi:10.1007/s11606-021-06737-1.

31. Sheehan DV, Lecrubier Y, Sheehan KH, et al. The Mini-International Neuropsychiatric Interview (M.I.N.I.): the development and validation of a structured diagnostic psychiatric interview for DSM-IV and ICD-10. *J Clin Psychiatry* 1998;59 Suppl 20:22-33.

32. Grönqvist H, Olsson EMG, Johansson B, et al. Fifteen challenges in establishing a multidisciplinary research program on eHealth research in a university setting: a case study. *J Med Internet Res* 2017;19(5):e173. doi:10.2196/jmir.7310.

For peer review only

33. Sjöström J, von Essen L, Grönqvist H. The origin and impact of ideals in eHealth research: experiences from the U-CARE research environment. *JMIR Res Protoc* 2014;3(2):e28. doi:10.2196/resprot.3202.
34. Craig P, Dieppe P, Macintyre S, et al. Developing and evaluating complex interventions: the new Medical Research Council guidance. BMJ 2008;337:a1655. doi:10.1136/bmj.a1655.
35. Ljungman L, Cernvall M, Grönqvist H, et al. Long-term positive and negative psychological late effects for parents of childhood cancer survivors: a systematic review. *PLoS One* 2014;9(7):e103340. doi:10.1371/journal.pone.0103340.
36. Carlsson T, Kukkola L, Ljungman L, et al. Psychological distress in parents of children treated for cancer: an explorative study. *PLoS One* 2019;14(6):e0218860. doi:10.1371/journal.pone.0218860.
37. Ljungman L, Boger M, Ander M, et al. Impressions that last: particularly negative and positive experiences reported by parents five years after the end of a child's successful cancer treatment or death. *PLoS One* 2016;11(6):e0157076. doi:10.1371/journal.pone.0157076.
38. Ljungman L, Cernvall M, Ghaderi A, et al. An open trial of individualized face-to-face cognitive behavior therapy for psychological distress in parents of children after end of treatment for childhood cancer including a cognitive behavioral conceptualization. *PeerJ* 2018;6:e4570. doi:10.7717/peerj.4570.
39. Wikman A, Kukkola L, Börjesson H, et al. Development of an internet-administered cognitive behavior therapy program (ENGAGE) for parents of children previously treated for cancer: Participatory action research approach. *J Med Internet Res* 2018;20(4):e133. doi:10.2196/jmir.9457.
40. Woodford J, Wikman A, Einhorn K, et al. Attitudes and preferences toward a hypothetical trial of an internet-administered psychological intervention for parents of children treated for cancer: web-based survey. *JMIR Ment Health* 2018;5(4):e10085. doi:10.2196/10085.
41. Barnes M, Wiles N, Morrison J, et al. Exploring patients' reasons for declining contact in a cognitive behavioural therapy randomised controlled trial in primary care. *Br J Gen Pract* 2012;62(598):e371-7. doi:10.3399/bjgp12X641492.
42. Farrand P, Woodford J, Llewellyn D, et al. Behavioural activation written self-help to improve mood, wellbeing and quality of life in people with dementia supported by informal carers (PROMOTE): a study protocol for a single-arm feasibility study. *Pilot Feasibility Stud* 2016;2:42. doi:10.1186/s40814-016-0083-x.
43. Sim J. Should treatment effects be estimated in pilot and feasibility studies? *Pilot Feasibility Stud* 2019;5:107. doi:10.1186/s40814-019-0493-7.
44. Sim J, Lewis M. The size of a pilot study for a clinical trial should be calculated in relation to considerations of precision and efficiency. *J Clin Epidemiol* 2012;65(3):301-8. doi:10.1016/j.jclinepi.2011.07.011.

For peer review only - http://bmjopen.bmj.com/site/about/guidelines.xhtml

45. Mbuagbaw L, Kosa SD, Lawson DO, et al. The reporting of progression criteria in protocols of pilot trials designed to assess the feasibility of main trials is insufficient: a meta-epidemiological study. *Pilot Feasibility Stud* 2019;5:120. doi:10.1186/s40814-019-0500-z.
46. Schulz KF, Altman DG, Moher D; et al. CONSORT 2010 statement: updated guidelines for reporting parallel group randomized trials. *Ann Intern Med* 2010;152(11):726-32. doi:10.7326/0003-4819-152-11-201006010-00232.
47. Elo S, Kyngäs H. The qualitative content analysis process. *J Adv Nurs* 2008;62(1):107-15. doi:10.1111/j.1365-2648.2007.04569.x.
48. Krefting L. Rigor in qualitative research: the assessment of trustworthiness. *Am J Occup Ther* 1991;45(3):214-22. doi:10.5014/ajot.45.3.214.
49. Miller CJ, Burgess JF Jr, Fischer EP, et al. Practical application of opt-out recruitment methods in two health services research studies. *BMC Med Res Methodol* 2017;17(1):57. doi:10.1186/s12874-017-0333-5.
50. Treweek S, Pitkethly M, Cook J, et al. Strategies to improve recruitment to randomised controlled trials. *Cochrane Database Syst Rev* 2010;(4):MR000013. doi:10.1002/14651858.MR000013.pub5.
51. Hunt KJ, Shlomo N, Addington-Hall J. Participant recruitment in sensitive surveys: a comparative trial of 'opt in' versus 'opt out' approaches. *BMC Med Res Methodol* 2013;13:3. doi:10.1186/1471-2288-13-3.
52. Khalifa SAM, Swilam MM, El-Wahed AAA, et al. Beyond the pandemic: COVID-19 pandemic changed the face of life. *Int J Environ Res Public Health* 2021;18(11):5645. doi:10.3390/ijerph18115645.
53. Adams EL, Smith D, Caccavale LJ, et al. Parents are stressed! Patterns of parent stress across COVID-19. *Front Psychiatry* 2021;12:626456. doi:10.3389/fpsyt.2021.626456.
54. Darlington AE, Morgan JE, Wagland R, et al. COVID-19 and children with cancer: parents' experiences, anxieties and support needs. *Pediatr Blood Cancer* 2021;68(2):e28790. doi: 10.1002/pbc.28790.
55. Klosky JL, Tyc VL, Lawford J, et al. Predictors of non-participation in a randomized intervention trial to reduce environmental tobacco smoke (ETS) exposure in pediatric cancer patients. *Pediatr Blood Cancer* 2009;52(5):644-9. doi:10.1002/pbc.21946.
56. Farrand P, Woodford J, Coumoundouros C, et al. Supported cognitive-behavioural therapy self-help versus treatment-as-usual for depressed informal caregivers of stroke survivors (CEDArS): feasibility randomised controlled trial. *Cogn Behav Therap* 2020;13:e23. doi:10.1017/S1754470X20000239
57. Ammari AB, Hendriksen C, Rydahl-Hansen S. Recruitment and reasons for non-participation in a family-coping-orientated palliative home care trial (FamCope). *J Psychosoc Oncol* 2015;33(6):655-74. doi:10.1080/07347332.2015.1082168.

For peer review only - http://bmjopen.bmj.com/site/about/guidelines.xhtml

58. Sandy LC, Glorioso TJ, Weinfurt K, et al. Leave me out: patients' characteristics and reasons for opting out of a pragmatic clinical trial involving medication adherence. *Medicine*. 2021;100(51):e28136. doi: 10.1097/MD.0000000000028136.

59. Vorderstrasse A, Reagan L, D'Eramo Melkus G, et al. Recruitment and enrollment of participants in an online diabetes self-management intervention in a virtual environment. Contemp Clin Trials. 2021;105:106399. doi: 10.1016/j.cct.2021.106399.

60. Bjertnaes O, Iversen HH, Skrivarhaug T. A randomized comparison of three data collection models for the measurement of parent experiences with diabetes outpatient care. *BMC Med Res Methodol* 2018;18(1):95. doi:10.1186/s12874-018-0557-z.

61. Shih T-H, Fan X. Comparing response rates from web and mail surveys: a meta-analysis. *Field Methods* 2008;20(3):249-271. doi:10.1177/1525822X08317085

62. Daikeler J, Bošnjak M, Lozar Manfreda K, Web versus other survey modes: an updated and extended meta-analysis comparing response rates. *J Surv Stat Methodol* 2020;8(3):513-539. doi:10.1093/jssam/smz008

63. Acquisti A, Brandimarte L, Loewenstein G. Secrets and likes: the drive for privacy and the difficulty of achieving it in the digital age. *J Consum Psychol* 2020;30(4):736-758. doi:10.1002/jcpy.1191

64. Robles T, Bordel B, Alcarria R, et al. Enabling trustworthy personal data protection in eHealth and well-being services through privacy-by-design. *Int J Distrib Sens Netw* 2020;16(5). doi:10.1177/1550147720912110.

65. Blatch-Jones A, Nuttall J, Bull A, Worswick L, Mullee M, Peveler R, Falk S, Tape N, Hinks J, Lane AJ, Wyatt JC, Griffiths G. Using digital tools in the recruitment and retention in randomised controlled trials: survey of UK Clinical Trial Units and a qualitative study. *Trials*. 2020;21(1):304. doi: 10.1186/s13063-020-04234-0.

66. Nanninga M, Reijneveld SA, Knorth EJ, et al. Expectations of barriers to psychosocial care: views of parents and adolescents in the community. *Eur Child Adolesc Psychiatry*. 2016;25(1):107-17. doi:10.1007/s00787-015-0717-1.

67. Zuidgeest M, Hendriks M, Koopman L, et al. A comparison of a postal survey and mixed-mode survey using a questionnaire on patients' experiences with breast care. *J Med Internet Res* 2011;13(3):e68. doi:10.2196/jmir.1241.

68. Alvarez-Galvez J, Salinas-Perez JA, Montagni I, et al. The persistence of digital divides in the use of health information: a comparative study in 28 European countries. *Int J Public Health* 2020;65(3):325-333. doi:10.1007/s00038-020-01363-w.

69. van Deursen AJ, van Dijk JA. Internet skills performance tests: are people ready for eHealth? *J Med Internet Res* 2011;13(2):e35. doi:10.2196/jmir.1581.

For peer review only - http://bmjopen.bmj.com/site/about/guidelines.xhtml

70. Mathiasen K, Riper H, Andersen TE, et al. Guided internet-based cognitive behavioral therapy for adult depression and anxiety in routine secondary care: observational study. *J Med Internet Res* 2018;20(11):e10927. doi:10.2196/10927.

71. Brown JSL, Murphy C, Kelly J, et al. How can we successfully recruit depressed people? Lessons learned in recruiting depressed participants to a multi-site trial of a brief depression intervention (the 'CLASSIC' trial). *Trials* 2019;20(1):131. doi:10.1186/s13063-018-3033-5.

72. Hughes-Morley A, Young B, Hempel RJ, et al. What can we learn from trial decliners about improving recruitment? Qualitative study. *Trials* 2016;17(1):494. doi:10.1186/s13063-016-1626-4.

73. van Warmerdam J, Zabih V, Kurdyak P, et al. Prevalence of anxiety, depression, and posttraumatic stress disorder in parents of children with cancer: a meta-analysis. *Pediatr Blood Cancer* 2019;66(6):e27677. doi:10.1002/pbc.27677.

74. Wikman A, Mattsson E, von Essen L, et al. Prevalence and predictors of symptoms of anxiety and depression, and comorbid symptoms of distress in parents of childhood cancer survivors and bereaved parents five years after end of treatment or a child's death. *Acta Oncol* 2018;57(7):950-957. doi:10.1080/0284186X.2018.1445286.

75. Badr H, Krebs P. A systematic review and meta-analysis of psychosocial interventions for couples coping with cancer. *Psychooncology* 2013;22(8):1688-704. doi:10.1002/pon.3200.

76. Heckel L, Gunn KM, Livingston PM. The challenges of recruiting cancer patient/caregiver dyads: informing randomized controlled trials. *BMC Med Res Methodol* 2018;18(1):146. doi:10.1186/s12874-018-0614-7.

77. Cuijpers P, Quero S, Noma H, et al. Psychotherapies for depression: a network meta-analysis covering efficacy, acceptability and long-term outcomes of all main treatment types. W*orld Psychiatry* 2021;20(2):283-293. doi:10.1002/wps.20860.

78. Robinson H, Appelbe D, Dodd S, et al. Methodological challenges in web-based trials: update and insights from the relatives' education and coping toolkit trial. *JMIR Ment Health* 2020;7(7):e15878. doi:10.2196/15878.

79. Brantnell A, Woodford J, Baraldi E, et al. Views of implementers and nonimplementers of internet-administered cognitive behavioral therapy for depression and anxiety: survey of primary care decision makers in Sweden. *J Med Internet Res* 2020;22(8):e18033. doi:10.2196/18033.

80. Gulliver A, Calear AL, Sunderland M, et al. Consumer-guided development of an engagement-facilitation intervention for increasing uptake and adherence for self-guided web-based mental health programs: focus groups and online evaluation survey. *JMIR Form Res* 2020;4(10):e22528. doi:10.2196/22528.

For peer review only

81. Nattinger AB, Pezzin LE, Sparapani RA, et al. Heightened attention to medical privacy: challenges for unbiased sample recruitment and a possible solution. *Am J Epidemiol* 2010;172(6):637-44. doi:10.1093/aje/kwq220.

82. Ward HJ, Cousens SN, Smith-Bathgate B, et al. Obstacles to conducting epidemiological research in the UK general population. *BMJ* 2004;329(7460):277-9. doi:10.1136/bmj.329.7460.277.

83. Halbesleben JR, Whitman MV. Evaluating survey quality in health services research: a decision framework for assessing nonresponse bias. *Health Serv Res* 2013;48(3):913-30. doi: 10.1111/1475-6773.12002.

For peer review only

For peer review only - http://bmjopen.bmj.com/site/about/guidelines.xhtml

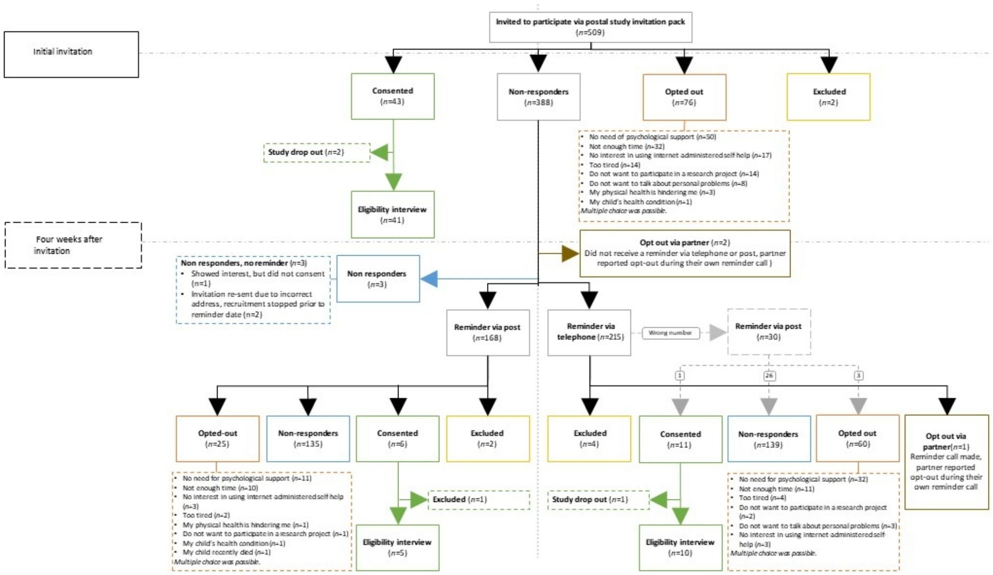

Figure 1. Recruitment flow via postal study invitation packs

87x50mm (300 x 300 DPI)

Checklist for Reporting Of Survey Studies (CROSS)

Section/topic	Item	Item description	Reported on page #
**Title and abstract**			
Title and abstract	1a	State the word "survey" along with a commonly used term in title or abstract to introduce the study's design.	1, 2
	1b	Provide an informative summary in the abstract, covering background, objectives, methods, findings/results, interpretation/discussion, and conclusions.	2
**Introduction**			
Background	2	Provide a background about the rationale of study, what has been previously done, and why this survey is needed.	4-5
Purpose/aim	3	Identify specific purposes, aims, goals, or objectives of the study.	5
**Methods**			
Study design	4	Specify the study design in the methods section with a commonly used term (e.g., cross-sectional or longitudinal).	5
Data collection methods	5a	Describe the questionnaire (e.g., number of sections, number of questions, number and names of instruments used).	7-8
	5b	Describe all questionnaire instruments that were used in the survey to measure particular concepts. Report target population, reported validity and reliability information, scoring/classification procedure, and reference links (if any).	N/A
	5c	Provide information on pretesting of the questionnaire, if performed (in the article or in an online supplement). Report the method of pretesting, number of times questionnaire was pre-tested, number and demographics of participants used for pretesting, and the level of similarity of demographics between pre-testing participants and sample population.	N/A
	5d	Questionnaire if possible, should be fully provided (in the article, or as appendices or as an online supplement).	N/A
Sample characteristics	6a	Describe the study population (i.e., background, locations, eligibility criteria for participant inclusion in survey, exclusion criteria).	5
	6b	Describe the sampling techniques used (e.g., single stage or multistage sampling, simple random sampling, stratified sampling, cluster sampling, convenience sampling). Specify the locations of sample participants whenever clustered sampling was applied.	6
	6c	Provide information on sample size, along with details of sample size calculation.	8
	6d	Describe how representative the sample is of the study population (or target population if possible), particularly for population-based surveys.	19
Survey	7a	Provide information on modes of questionnaire administration, including the type and number of contacts, the location where the survey was conducted (e.g., outpatient	6

For peer review only

administration		room or by use of online tools, such as SurveyMonkey).	
	7b	Provide information of survey's time frame, such as periods of recruitment, exposure, and follow-up days.	9
	7c	Provide information on the entry process:<br>–>For non-web-based surveys, provide approaches to minimize human error in data entry.<br>–>For web-based surveys, provide approaches to prevent "multiple participation" of participants.	N/A
Study preparation	8	Describe any preparation process before conducting the survey (e.g., interviewers' training process, advertising the survey).	N/A
Ethical considerations	9a	Provide information on ethical approval for the survey if obtained, including informed consent, institutional review board [IRB] approval, Helsinki declaration, and good clinical practice [GCP] declaration (as appropriate).	18
	9b	Provide information about survey anonymity and confidentiality and describe what mechanisms were used to protect unauthorized access.	6
Statistical analysis	10a	Describe statistical methods and analytical approach. Report the statistical software that was used for data analysis.	8
	10b	Report any modification of variables used in the analysis, along with reference (if available).	N/A
	10c	Report details about how missing data was handled. Include rate of missing items, missing data mechanism (i.e., missing completely at random [MCAR], missing at random [MAR] or missing not at random [MNAR]) and methods used to deal with missing data (e.g., multiple imputation).	N/A
	10d	State how non-response error was addressed.	N/A
	10e	For longitudinal surveys, state how loss to follow-up was addressed.	N/A
	10f	Indicate whether any methods such as weighting of items or propensity scores have been used to adjust for non-representativeness of the sample.	N/A
	10g	Describe any sensitivity analysis conducted.	N/A
**Results**			
Respondent characteristics	11a	Report numbers of individuals at each stage of the study. Consider using a flow diagram, if possible.	9-10
	11b	Provide reasons for non-participation at each stage, if possible.	11-15
	11c	Report response rate, present the definition of response rate or the formula used to calculate response rate.	9

For peer review only

	11d	Provide information to define how unique visitors are determined. Report number of unique visitors along with relevant proportions (e.g., view proportion, participation proportion, completion proportion).	N/A
Descriptive results	12	Provide characteristics of study participants, as well as information on potential confounders and assessed outcomes.	10-11
Main findings	13a	Give unadjusted estimates and, if applicable, confounder-adjusted estimates along with 95% confidence intervals and p-values.	11
	13b	For multivariable analysis, provide information on the model building process, model fit statistics, and model assumptions (as appropriate).	N/A
	13c	Provide details about any sensitivity analysis performed. If there are considerable amount of missing data, report sensitivity analyses comparing the results of complete cases with that of the imputed dataset (if possible).	N/A
**Discussion**			
Limitations	14	Discuss the limitations of the study, considering sources of potential biases and imprecisions, such as non-representativeness of sample, study design, important uncontrolled confounders.	19
Interpretations	15	Give a cautious overall interpretation of results, based on potential biases and imprecisions and suggest areas for future research.	15-18
Generalizability	16	Discuss the external validity of the results.	19
**Other sections**			
Role of funding source	17	State whether any funding organization has had any roles in the survey's design, implementation, and analysis.	20
Conflict of interest	18	Declare any potential conflict of interest.	20
Acknowledgements	19	Provide names of organizations/persons that are acknowledged along with their contribution to the research.	20

For peer review only
